# Metallomics in deep time and the influence of ocean chemistry on the metabolic landscapes of Earth’s earliest ecosystems

**DOI:** 10.1038/s41598-020-61774-w

**Published:** 2020-03-18

**Authors:** Keyron Hickman-Lewis, Barbara Cavalazzi, Stéphanie Sorieul, Pascale Gautret, Frédéric Foucher, Martin J. Whitehouse, Heejin Jeon, Thomas Georgelin, Charles S. Cockell, Frances Westall

**Affiliations:** 1 CNRS Centre de Biophysique Moléculaire UPR 4301, Rue Charles Sadron, CS80054, 45071 Orléans, France; 2 Dipartimento di Scienze Biologiche, Geologiche e Ambientali (BiGeA), Università di Bologna, Via Zamboni 67, I-40126 Bologna, Italy; 3 Department of Geology, University of Johannesburg, PO Box 524, Auckland Park 2006, Johannesburg, South Africa; 4 University of Bordeaux, CNRS, IN2P3, CENBG, 19 Chemin du Solarium, 33175 Gradignan, France; 5 Université d’Orléans, ISTO, UMR 7327, 45071, Orléans, France; CNRS, ISTO, UMR 7327, 45071 Orléans, France; BRGM, ISTO, UMR 7327, BP 36009, 45060 Orléans, France; 6 Department of Geosciences, Swedish Museum of Natural History, Box 50007, SE-104 05 Stockholm, Sweden; 7 Sorbonne Universités, UPMC Paris 06, CNRS UMR 7197, Laboratoire de Réactivité de Surface, 4 Place Jussieu, 75005 Paris, France; 8 UK Centre for Astrobiology, School of Physics and Astronomy, University of Edinburgh, James Clerk Maxwell Building, Edinburgh, EH9 3JZ United Kingdom

**Keywords:** Geochemistry, Evolutionary theory, Palaeontology, Biogeochemistry, Palaeoecology

## Abstract

Modern biological dependency on trace elements is proposed to be a consequence of their enrichment in the habitats of early life together with Earth’s evolving physicochemical conditions; the resulting metallic biological complement is termed the metallome. Herein, we detail a protocol for describing metallomes in deep time, with applications to the earliest fossil record. Our approach extends the metallome record by more than 3 Ga and provides a novel, non-destructive method of estimating biogenicity in the absence of cellular preservation. Using microbeam particle-induced X-ray emission (µPIXE), we spatially quantify transition metals and metalloids within organic material from 3.33 billion-year-old cherts of the Barberton greenstone belt, and demonstrate that elements key to anaerobic prokaryotic molecular nanomachines, including Fe, V, Ni, As and Co, are enriched within carbonaceous material. Moreover, Mo and Zn, likely incorporated into enzymes only after the Great Oxygenation Event, are either absent or present at concentrations below the limit of detection of µPIXE, suggesting minor biological utilisation in this environmental setting. Scanning and transmission electron microscopy demonstrates that metal enrichments do not arise from accumulation in nanomineral phases and thus unambiguously reflect the primary composition of the carbonaceous material. This carbonaceous material also has δ^13^C between −41.3‰ and 0.03‰, dominantly −21.0‰ to −11.5‰, consistent with biological fractionation and mostly within a restricted range inconsistent with abiotic processes. Considering spatially quantified trace metal enrichments and negative δ^13^C fractionations together, we propose that, although lacking cellular preservation, this organic material has biological origins and, moreover, that its precursor metabolism may be estimated from the fossilised “palaeo-metallome”. Enriched Fe, V, Ni and Co, together with petrographic context, suggests that this kerogen reflects the remnants of a lithotrophic or organotrophic consortium cycling methane or nitrogen. Palaeo-metallome compositions could be used to deduce the metabolic networks of Earth’s earliest ecosystems and, potentially, as a biosignature for evaluating the origin of preserved organic materials found on Mars.

## Introduction

“*The system of cell chemistry…cannot be divorced from the environment any more than it can be separated from a code. All the basic chemicals and energy come from the environment and this remains true to this day*”

R. J. P. Williams and J. J. R Fraústo da Silva (*Journal of Theoretical Biology*, 2003).

A wide range of carbonaceous materials (CM) occur in Palaeoarchaean (~3.6-3.2 Ga) rocks. Some CM is demonstrably biogenic^1,2^, however, due to maturation, metamorphism and the difficulty inherent in distinguishing between CM derived from biogenic and abiotic processes, the origin of most Archaean CM remains controversial^3,4^. This is further compounded by the fact that both biogenic and abiotic CM would have been generated in early habitable environments, for example, hydrothermally influenced oceans with significant exogenous input^1,4–6^. As a result, the fossil record of early life, and especially the potential fossil record encoded in Palaeoarchaean CM, remains incompletely understood^3,7^. Nonetheless, similarities in the occurrence, morphology and chemistry of CM from similar settings should support common origins. Generally, abiotic processes are more likely to form CM with an unexceptional continuum of geological-biogeochemical characteristics, whereas more striking patterns with restricted ranges in chemical characteristics are expected in biogenic CM in spite of subsequent alteration^2,8–10^. Such patterns, when discerned in Archaean CM, usually have the hallmarks of metamorphosed biological material in terms of C-N-S isotope geochemistry, spectral characteristics from, e.g., Raman and IR spectroscopy, and the spatial correlation of CHNOPS elements, albeit muted compared to bio-indicative signals in extant biomass^2,5,8,10,11^. Quantitative approaches to characterising biogeochemistry in deep time are therefore of great value in ameliorating our understanding of Precambrian palaeoecology. In the case of unambiguously biogenic CM, for example microbial mat laminations, repeated similarities in isotopic, spectral or molecular characteristics might indicate the metabolic affinities of precursor biomass (e.g.^9,10,12,13^). Regrettably, although carbon isotope ratios related to microfossils and organo-sedimentary structures can be interpreted as the effects of organisms or consortia^9,12,14^, δ^13^C values mostly between −40‰ and −10‰ reported for Archaean “biomass” are consistent with multiple biological pathways, for example, anoxygenic photosynthesis, sulphate-reduction, photoferrotrophy and methanogenesis^15–17^. Furthermore, these values fall within the range exhibited by the products of abiotic Fischer-Tropsch-type processes (e.g.^18^), which were widespread in the environments of early life.

**Figure 1:**
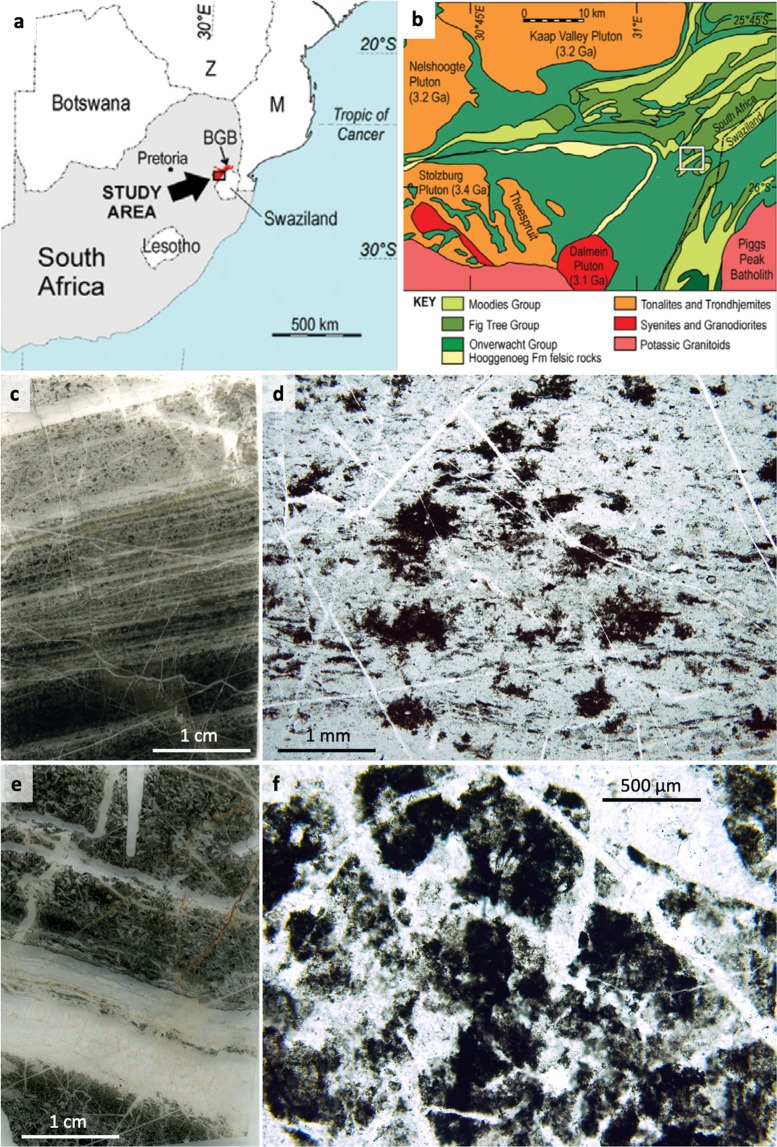
Sampling location maps (**a,b**) and petrographic context of the microstructures of interest; irregular clots (**c,d**) and volcanic particle coatings (**e,f**) sourced from the 3.33 Ga Josefsdal Chert (Barberton greenstone belt, South Africa). (**a**) Map of southern Africa showing the location of the Barberton greenstone belt. (**b**) Map of the southern portion of the Barberton greenstone belt; white box indicates the location of the Josefsdal Chert, near the border of South Africa and Eswatini, from which all studied samples were collected. (**c**) Thin section of weakly laminated clotted carbonaceous chert corresponding to black massive chemical chert observed in the field (see Fig. S2). Carbonaceous clots are distributed throughout a silica matrix; other phases include scattered microminerals including pyrite and chromite spinel (see Fig. 2). (**d**) High-resolution photomicrograph showing irregular, stellate carbonaceous clots of up to ~1 mm diameter. (**e**) Thin section of laminated black and white banded chert corresponding to laminated chert observed in the field (see Fig. S2). (**f**) High-resolution photomicrograph showing sub-rounded and sub-angular pseudomorphed and silicified volcanic particles coated and impregnated with carbonaceous material (dark regions).

We here further the range of biogeochemical proxies for decoding palaeoecology by proposing that CM of otherwise enigmatic origins (Fig. 1) could, when preserving specific trace element enrichments, be diagnostic of the metabolism of precursor biomass. We term this trace element signature the “palaeo-metallome”, in recognition of the fact that it represents a remnant of chemical selectivity by cells leading to their inorganic elemental composition (the “metallome”)^19,20^. Metals facilitate numerous biosynthetic processes as parts of oxidoreductases and other metalloenzymatic and proteinic nanomachines, in addition to being bound by extracellular polymers (e.g.^20–23^). Decoding patterns of metal enrichment in fossil biomass, historically difficult due to limits of instrumental resolution and limited understanding of the rapidity of silicification processes, may be achieved using quantified ion-beam analyses at high resolution. Although microbial material is capable of metal retention even after cell death^22,24,25^, early and post-diagenetic effects in rocks with poor preservation potential may alter elemental compositions *via* leaching and fluid remobilisation^22,24^. Very rapidly silicified, exceptionally preserved CM that may be able to preserve relic bio-accumulation of trace metals without post-diagenetic alteration is, however, common in Archaean chert horizons^1,4,5^.

## Rationale: A Framework for Estimating the Palaeo-Metallome of Fossil Biomass

The metallome is the component of biosynthetic chemistry comprising the metals and metalloids of a biological system, i.e., the inorganic complement to the proteome and genome^19–21,26^. In palaeobiological terms, the metallome is best appraised as the totality of elements within cellularly derived fossils. Currently, the oldest metallomes date from 131-120 Ma feathers^27^ and 50 Ma leaves^28^, but it has nonetheless been suggested that linking metal signatures from fossils in deep time to their organic chemistry may illuminate metallome evolution throughout geological history^29,30^. The relative proportions of elements in well-preserved CM may also be linked to their presence in proteins, metabolites and other biomolecules within the predicted biological system^26^. A palaeo-metallomic biosignature in the rock record should therefore chart systematic, bioaccumulative interactions between organisms and the external environmental compartment sourcing the elements that constitute the metallome^21^.

If modern biological dependency upon trace metals is a consequence of the richness of these elements in the environments of early life^20,21^, proposing a palaeo-metallomic signature in deep time demands a framework of palaeoenvironmental reconstruction. The 3.33 Ga Josefsdal Chert (JC; equivalent to Kromberg Formation unit K3c, Barberton greenstone belt; Figs. 1a,b, S1,S2), from which our samples were collected, is best represented by outcrops between Ekulindeni, Mpumalanga province, South Africa, and Bulembu, Hhohho region, Eswatini. The JC is an archetypal Palaeoarchaean chert comprising silicified volcanogenic sediments intercalated with near-pure chert horizons and chemical sediments. These horizons contain variably preserved microbial vestiges in the forms of microfossils and microbial mats^31,32^. The three-dimensional preservation of such biosignatures^5^ stands testament to their rapid silicification, which necessarily commenced during the life cycle of the organism^33^. Moreover, the JC presents copious outcrop and geochemical evidence for widespread hydrothermally influenced sedimentary fabrics^31,32^; Fig. S2). The organisms inhabiting such environments are estimated to have been (hyper)thermophilic and, if immediately downstream of hydrothermal effusions, likely chemosynthetic with polyextremotolerance to salinity and biotoxic metal enrichments^14,31,33–35^. Regrettably, the chemosynthetic biosphere leaves enigmatic vestiges of its existence in the rock record, beset by simple morphologies and subsequent alteration by diagenetic and metamorphic processes even where rapidly preserved by silicification^14,31,33^. The Palaeoarchaean fossil record nonetheless presents an ideal window on the primitive thermophilic biosphere: it dates from a time when life on Earth was driven dominantly by internal heat^35–39^. Both chemotrophic and phototrophic metabolisms have been predicted as early as 3.8 Ga^11,12,15,35,40^ and the probability of chemosynthetic inhabitants in the palaeoenvironments represented by Archaean cherts makes these rocks ideal text cases for palaeo-metallomics, since such extremotolerant thermophiles have unique metallome compositions with specific elemental requirements^41^.

Following palaeoenvironmental reconstruction, deducing palaeo-metallomic signatures demands petrographic and geochemical demonstration that the CM studied is of biogenic origin. This entails proof of CM syngenicity, ideally using high-magnification optical petrography coupled to Raman spectroscopy and geothermometry, and carbon isotope geochemistry to indicate negative δ^13^C values consistent with biological fractionation.

Deducing metallomic biosignatures in Precambrian rocks (the main focus of this study) demands the detection of metal and metalloid signatures within CM, and will pose very different challenges to the determination of metallomes in modern and sub-fossil samples. Although it should be possible to calculate the elemental composition of CM when present in sufficiently high concentrations, linking elemental concentrations to specific functions within the genome or proteome is impossible due to the lack of preservation of biomolecules from the genome or proteome in ancient fossils^27,42^. We propose that it is more prudent to consider a conservative approach akin to that of Phanerozoic metallome studies involving the quantification of the spatial distribution of trace metals within primary structures of plausible biological origin followed by an attempt to logically link these metal enrichments to biosynthetic pathways possible within the constraints of the palaeoenvironment^26–28^. Furthermore, ancient samples, particularly often-controversial carbonaceous materials in Archaean cherts, require proof that post-diagenetic processes did not contribute to the eventual signal^22^ or, where present, that such overprints can be quantified.

## Results: Testing the Palaeo-Metallome Hypothesis

### Step I: Palaeoenvironmental reconstruction

**Figure 2:**
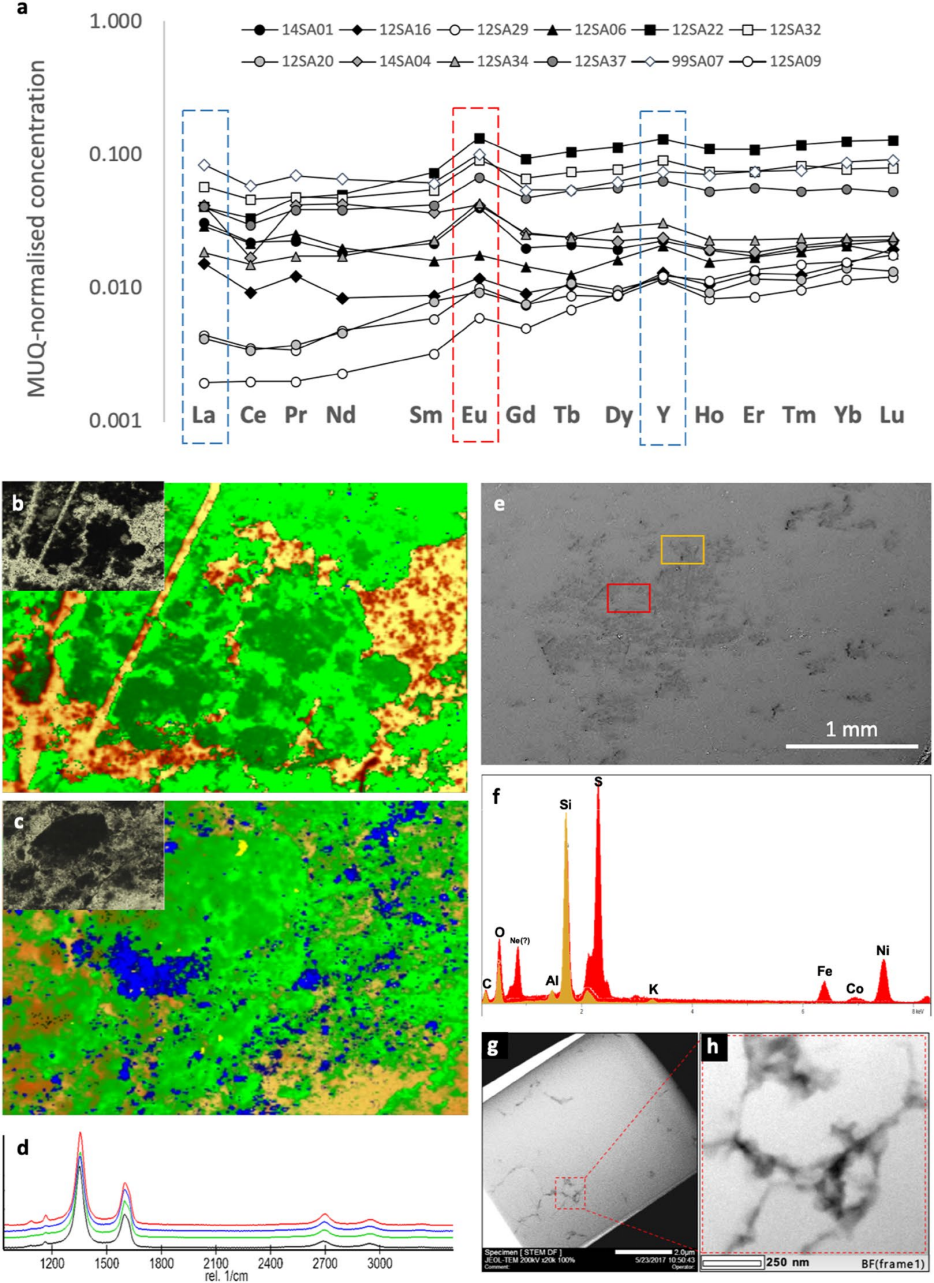
Selected geochemical analyses relevant to the palaeoenvironmental characterisation of the 3.33 Ga Josefsdal Chert. (**a**) REE+Y compositions of various samples from the studied horizons of the Josefsdal Chert normalised to Mud from Queensland (MuQ), a mixed mafic-felsic shale estimate of Archaean continental composition. HREE enrichment and positive La and Y anomalies indicates hydrogenous, thalassogenic contributions (marine), whereas positive Eu anomalies indicate hydrothermal fluid contributions. (**b**,**c**) Raman spectroscopy mapping of a representative irregular clot (**b**) and carbon-impregnated volcanic particle (**c**). Inset optical images show the regions of analysis; field of view = 1 mm. Green = carbonaceous material; yellow-orange = quartz; blue = anatase. Anatase can be considered a proxy for the alteration of volcanic particles associated with carbonaceous material. (**d**) Average Raman spectra for carbonaceous material in four studied samples (99SA07, black; 12SA09, green; 12SA16, blue; 14SA01, red). (**e**,**f**) SEM-EDS analyses of non-silicified mineral phases associated with carbonaceous material. Spectra are colour-correspondent to points within the regions of analysis shown. Red spectra = Ni-rich pyrite; beige spectra = K-Al phyllosilicate. Extensive SEM-EDS analyses are shown in Figs. S4-S8. (**g,h**) TEM micrographs from within clotted microstructures (sample 99SA07) showing the discontinuous distribution of carbonaceous material (black) within the microquartz matrix (grey).

**Figure 3:**
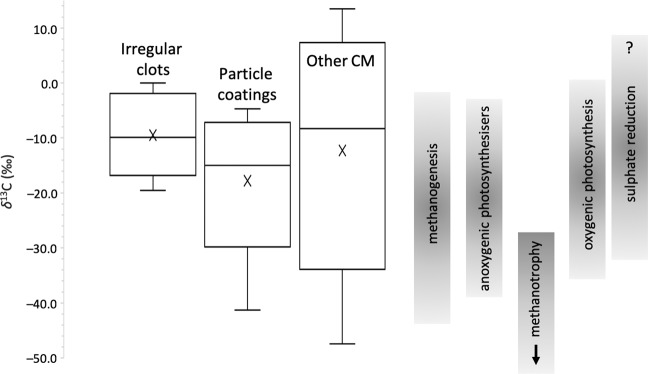
Box-plot representations of SIMS δ^13^C data for irregular clots, particle coatings/impregnations and generic carbonaceous material (other CM) in the studied samples of the Josefsdal Chert. Comparisons are given with well-defined carbon isotope fractionations for the products of anoxygenic and oxygenic photosynthesisers, and methane and sulphur-cycling organisms (compiled after McCandless and Gurney^77^; Schidlowski^15^; Londry and Des Marais^76^; Vieth and Wilkes^16^). Well-defined microstructures (clots and coatings) have negative and restricted δ^13^C ranges consistent with biological mediation by multiple pathways. Coatings are characterised by δ^13^C values slightly more negative than clots (averaging −15.16‰ versus −9.44‰). Generic CM (other CM) in the same samples is characterised by a wide range of positive and negative δ^13^C values (between −47.4 ± 8.3‰ and +13.5 ± 2.0‰) bearing less resemblance to products resulting from specific biological pathways.

The “time capsule” preservation of morphological and geochemical characteristics afforded by silicification, i.e. rapid encapsulation, lithification, and close to zero post-diagenetic alteration, is a unique taphonomic setting^43^. The extremely rapid silicification characterising Archaean cherts results from a combination of the high (oversaturated-supersaturated) Si content of the Archaean oceans due to both dissolution of seafloor volcanics and the lack of biological uptake of silica into tests and shells with respect to the modern, coupled with Si-rich hydrothermal effluence, which was demonstrably more significant on the Archaean Earth than at present^31,34^. The resulting rapidity of preservation assures that pristine and unaltered geochemical signatures are retained (Fig. 2), for example REE+Y compositions defining palaeodepositional conditions (Fig. 2a), Raman spectral characteristics demonstrating that the thermal maturity of this disordered CM is commensurate with that of the host rock (Figs. 2b–d, S3), minerals and materials resistant to silicification (Fig. 2e–h) and isotopic compositions evidencing fractionation pathways in the precursor materials (Fig. 3).

Normalised REE+Y patterns (measured using ICP-MS) are characterised by fractionated trends with weak HREE enrichment ((Pr/Yb)_MUQ_ = 0.57 to 2.41), mostly positive La anomalies [La/La*_MUQ_ = La_MUQ_/(Pr*_MUQ_(Pr_MUQ_/Nd_MUQ_)^2^)] between 0.93 and 2.90, the absence of negative Ce anomalies, positive Eu anomalies [Eu/Eu*_MUQ_ = Eu_MUQ_/(Sm^2^_MUQ_ * Tb_MUQ_)^1/3^] between 1.01 and 2.02, and positive Y anomalies [Y/Y*_MUQ_ = Y_MUQ_/(0.5Er_MUQ_ * 0.5Ho_MUQ_)] between 1.13 and 1.45 (i.e., superchondritic Y/Ho ratios between 30.31 and 38.57). La, Y, Y/Ho and Eu systematics indicate that the palaeodepositional setting of these cherts was influenced by marine and hydrothermal fluids^44^, with muted anomaly characteristics relative to pure marine precipitates indicating basin restriction^45^ and increased terrigenous influence^46^, likely from exposed mafic-felsic landmasses. High concentrations of Fe- and potentially Mn-oxyhydroxides in semi-restricted disequilibrium settings such as this would have stimulated the concentration of trace and rare earth elements through their induced dissolution, enhancing the local bioavailability of these elements^44,46^. The environment of the Josefsdal Chert was therefore metal-rich, warm-hot, anoxic, and replete with chemical disequilibria as a result of the confluence of discrete fluid reservoirs. The studied samples thus reflect a habitable environment that seems to have been typical on the early Earth (see Discussion of^38^).

### Step II: Syngenicity and biogenicity of CM

Two petrographically distinct morphologies of CM were studied using secondary ion mass spectrometry and microbeam particle-induced X-ray emission (µPIXE): i) irregular, stellate clots of 200–2,000 µm diameter (Fig. 1c,d) and ii) non-isopachous coatings on altered volcanic particles (Fig. 1e,f). Both of these microstructures occur within the same millimetre-scale domains as the well-preserved microbial mats and biofilms in the JC, i.e., within the black bands of black-and-white banded cherts and within carbon-bearing regions of massive black cherts (Figs. 1, S2). Microbial mats either drape over clotted textures or coexist with them in the form of incompletely formed biofilm fragments. The studied clots are ‘free-floating’ microstructures in volcano-hydrothermal sediments, co-occurring with degraded microbial mats, and were interpreted as putative degraded chemosynthetic biomass growing *in situ* within a gel-like sediment by Westall *et al*.^31^. Archaean clotted carbonaceous material was first ascribed a potential biological origin by Walsh^47^, who compared it to microbe-mineral aggregates in modern marine environments^48,49^. Coating-like fabrics on volcanic particles were interpreted as putative lithotrophic coverings of specific volcanogenic fragments based on similarity to known microfossils (cf.^5,31^). Neither case for biogenicity has yet been unambiguously demonstrated, however, there are many very strong arguments against these CM morphologies being abiotic in origin, the full reasoning for which is explained in the Supplementary Material. In brief: (i) non-isopachous, irregular morphologies argue against an origin as condensation fabrics seen in contemporaneous CM^50^; (ii) the lack of continuous, blanket-like carbonaceous laminae with grain-supported textures argues against an origin as either meteoritic^6^ or detrital, sequestered CM (see^13^); (iii) no aggregates with radioactive cores have been identified, therefore an origin as pyrobitumen^51^ is not possible; (iv) no CM-infused tephra-like morphologies^52^ have been observed; and (v) the restricted range of δ^13^C is inconsistent with FTT-derived hydrocarbons^18,53^. Thus, no plausible abiological interpretation for either the irregular clot or particle coating CM morphologies is sustained under scrutiny. Conversely, a biological origin is strongly supported by palaeoenvironmental and petrological context^31,32^ (Fig. 1), for example that laminated and clotted CM alike would have been able to grow through attachment on, or matrix-supported suspension within, a gel-like Si-rich volcanogenic/chemical sediment. Although previous studies e.g.^1,47^ have inferred that there may be a genetic link between mats and clots, there is no evidence for this in our samples. Specifically, since mats and clots occur within the same horizons without evidence for differential compression, the two must have developed simultaneously.

Raman spectra are qualitatively characteristic of disordered carbon with a broad D band at 1,350 cm^−1^ and minor G band at 1,580 cm^−1^. Coupled with peak metamorphic temperatures of between 285 °C and 298 °C estimated according to the Raman geothermometer of Kouketsu *et al*.^54^, these spectra indicate that all studied CM has a thermal history consistent with that of the host rock (Fig. 2b–d), i.e., the CM is syngenetic. The studied microstructures have δ^13^C compositions between −41.3‰ and +0.03‰, averaging −9.44‰ and −15.16‰ for clots and coatings, respectively (Fig. 3; Table S1), consistent with biological fractionation by multiple pathways as reported in contemporaneous rocks (e.g.^9,15–17^ and references therein). δ^13^C values are slightly more negative in coatings than clots. Both microstructures are characterised by a restricted range of δ^13^C values compared to generic carbonaceous fragments and particles of uncertain origins (i.e., abiotic or biotic) in the same samples (Fig. 3; Table S1). Since the biogenicity of these microstructures is probable, it becomes justifiable to calculate the trace element composition of this CM in the framework of the palaeo-metallome.

### Step III: Trace element signatures in CM

µPIXE was performed at the microbeam beamline of the AIFIRA facility described in Sorieul *et al*.^55^. Multiple examples of each CM morphology were measured. Their adjacent matrices i.e., a relic of the palaeodepositional fluid, were used as the ambient concentration against which absolute elemental concentrations within specific microstructures were compared. Multiple analyses of the matrix were conducted in each sample, providing average matrix values for elemental concentration that were found to be indistinguishable from bulk rock ICP-MS measurements (Figs. S10-S11; Table S3), i.e. ICP-MS and µPIXE results are, as expected, directly comparable. µPIXE was used to map elements in the mass range from P to beyond Mo with ppm accuracy^56,57^.

**Figure 4:**
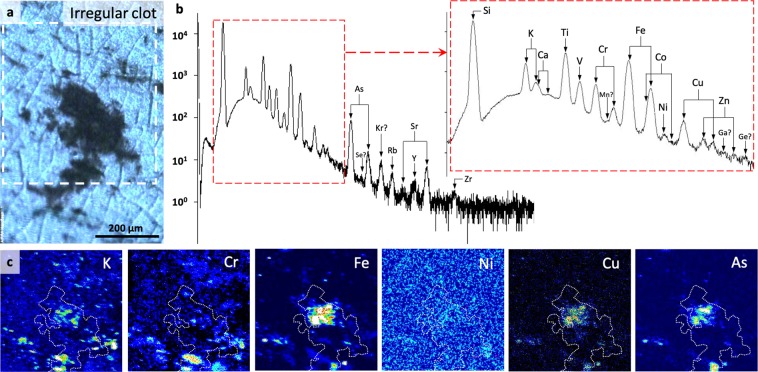
µPIXE spatially quantified elemental mapping within irregular clots. (**a**) µPIXE optical image of representative irregular clot. White box indicates the region for which the spectrum (**b**) and element maps (**c**) are given. (**b**) µPIXE spectrum with peak identification for the energy range 0–18 keV. (**c**) Element heat maps (black = low concentration, white = high concentration) for the region indicated in A, showing localised significant enrichments corresponding to entrained micromineral particles such as those identified by SEM-EDS in Fig. 2, but also a general enrichment within carbonaceous material (outlined by dotted white line) relative to the matrix.

Excluding enrichments due to micromineral phases (which should not be taken as a part of the metallomic signature, since it is usually not possible to distinguish microbially mediated and abiotic precipitates from elemental fractionation and concentration alone^58^), we found that irregular clots exhibit significant (>150% relative to matrix concentrations) enrichments (e.g., Figs. 4a–c, S12) in nine bio-functional elements: P, S, Ti, V, Fe, Co, Ni and As. Furthermore, Mn, Cu, Zr and Mo appear modestly enriched (100–150%) relative to the matrix, although all of these and Zn occur at concentrations below the limit of detection appropriate for quantification (Table S2). Ca and Cr are depleted (<100%) relative to the matrix. Although P is depleted relative to the matrix in two analyses, high densities of apatite (Ca_5_(PO_4_)_3_) crystals were observed in the adjacent matrix, and P concentrations within CM are of the same order of magnitude as in analyses where CM is enriched. Average elemental concentrations in irregular clots follow the trend: K > Ti > Fe, P > V > Ni > Cr > As, S > Co > *Cu* > *Zn* > *Zr, Mo, Mn*. Elements listed in italics were mapped and are thus present but at concentrations below the theoretical limit of quantification. Anomalously high S concentrations in some analyses are attributable to micro-pyrite grains that are intimately associated with CM. Such morphologies and occurrences of pyrite are consistent with microbial metabolism, for example after degradation of biogenic organic material by sulphate-reducing bacteria or the anaerobic oxidation of methane (e.g.^14,59,60^). Although micro-pyrites identified in SEM imaging are point sources for As, Ni and Cu (≤11,500 ppm), these elements are also broadly and diffusely distributed throughout CM in irregular clots (Figs. 4, S12).

**Figure 5:**
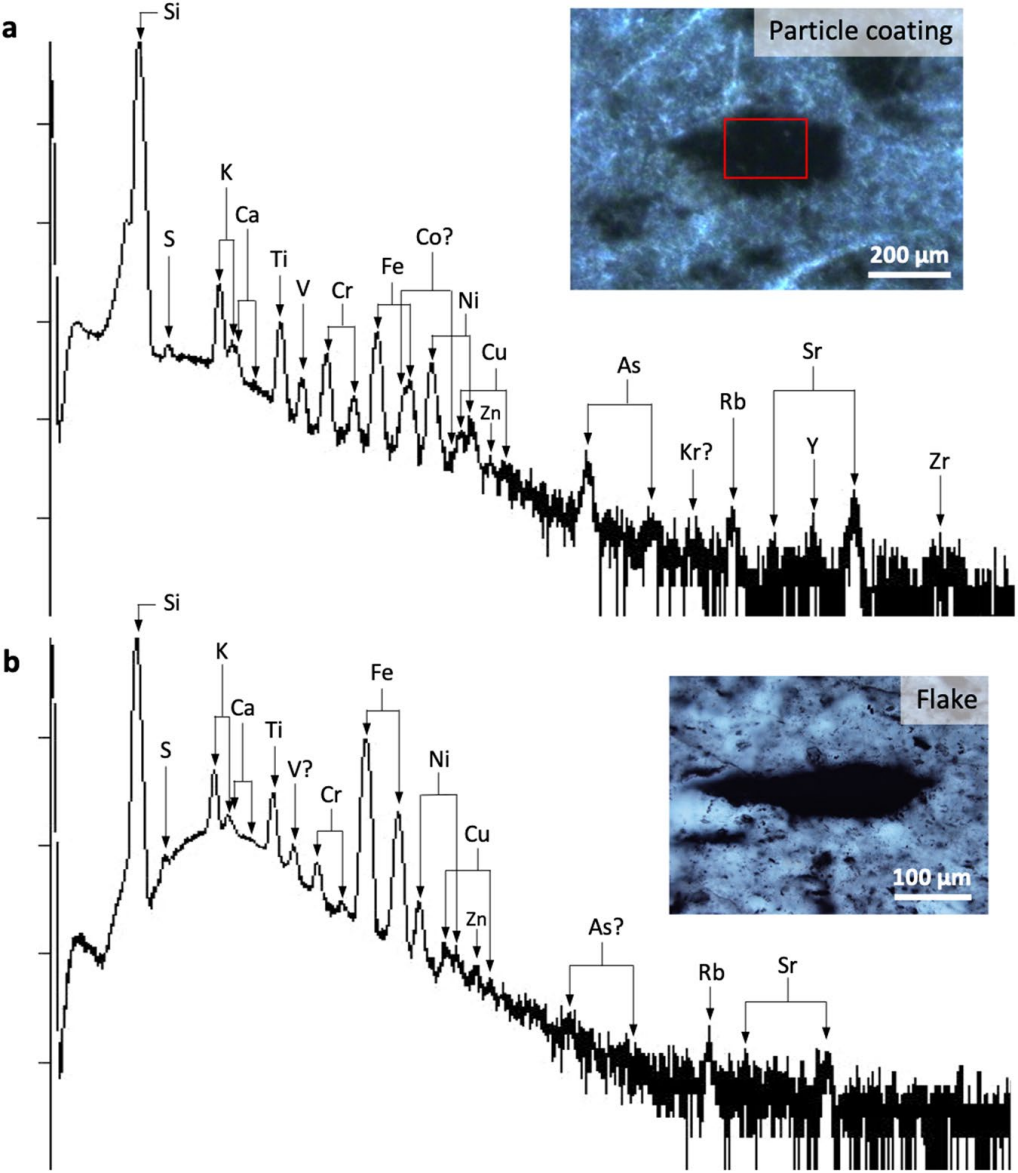
µPIXE spectra after mapping within carbon-rich volcanic particle coatings (**a**) and generic carbonaceous material in the form of flakes (**b**). (**a**) µPIXE optical image of representative particle coating and accompanying spectra with peak identification for the energy range 0–18 keV. (**b**) µPIXE optical image of a representative carbonaceous flake and accompanying spectra with peak identification for the energy range 0–18 keV. Note the very strong enrichments in certain transition metals within the flake (Fe, Ni, Ti) corresponding to their spatially restricted accumulation within micro-mineral particles as shown in Fig. S15-S16.

Coatings of CM on volcanic particles show similar elemental enrichments to clots, but with inconsistent P distributions. Coatings are significantly enriched relative to the matrix in S, V, Ni, Cu, As and Zr, although Zn concentrations are below the limit of detection for quantification (Figs. 5a, S13). K, Fe and Co are modestly enriched, whereas P, Ca, Ti and Cr are depleted. Mn, Zn and Mo are present at concentrations below the limit of detection for quantification. Anomalously high Ti and Cu values in some analyses may results from microscopic ilmenite (into which Cu can substitute), titanomagnetite and anatase intermixed with volcanogenic material and CM (identified *via* SEM-EDS and Raman spectroscopy) or from accumulation associated with biotoxicity (after^33^). Average elemental concentrations in clots follow the trend: K, Ni > Cu, Fe > V > S > As > P, Ti > Co > Zr > Ca > *Zn, Mo, Mn*. In both irregular clots and particle coatings, a trend in decreasing absolute concentration of trace elements with atomic mass is observed, whereas enrichment relative to the matrix is element-dependent (Figs. 5a, S13, Table S2).

Generic flakes, particles and fragments of CM throughout rocks of the same cherts that do not conform to any specific microstructure (e.g., Figs. 5b, S14) were analysed as a comparison to the clot and coating microstructures. Although there is again a decreasing absolute enrichment with atomic mass (Fig. S15, Table S2), specific elemental enrichments are either minor or inexistent relative to the matrix (Fig. 5b). High-resolution µPIXE mapping shows that transition metal enrichments are invariably linked to microminerals in generic CM (Fig. S15–16). Discounting microminerals, the trend of elemental enrichments in generic flakes of CM is indistinguishable from that of the matrix, and thus very different in both magnitude and distribution to the enrichments measured in irregular clots and particle coatings.

## Microstructure-Specific Trace Element Signatures

Clot and coating morphologies are characterised by similar negative δ^13^C values, albeit with slightly more negative values in coatings (centred on −15.16‰) than in clots (centred on −9.44‰). Flakes show a wide range of δ^13^C (between −47.4 ± 8.3‰ and +13.5 ± 2.0‰) including some positive values (Fig. 3). Clotted and particle coating morphologies are therefore characterised by restricted carbon isotope fractionations more indicative of biological processing.

**Figure 6:**
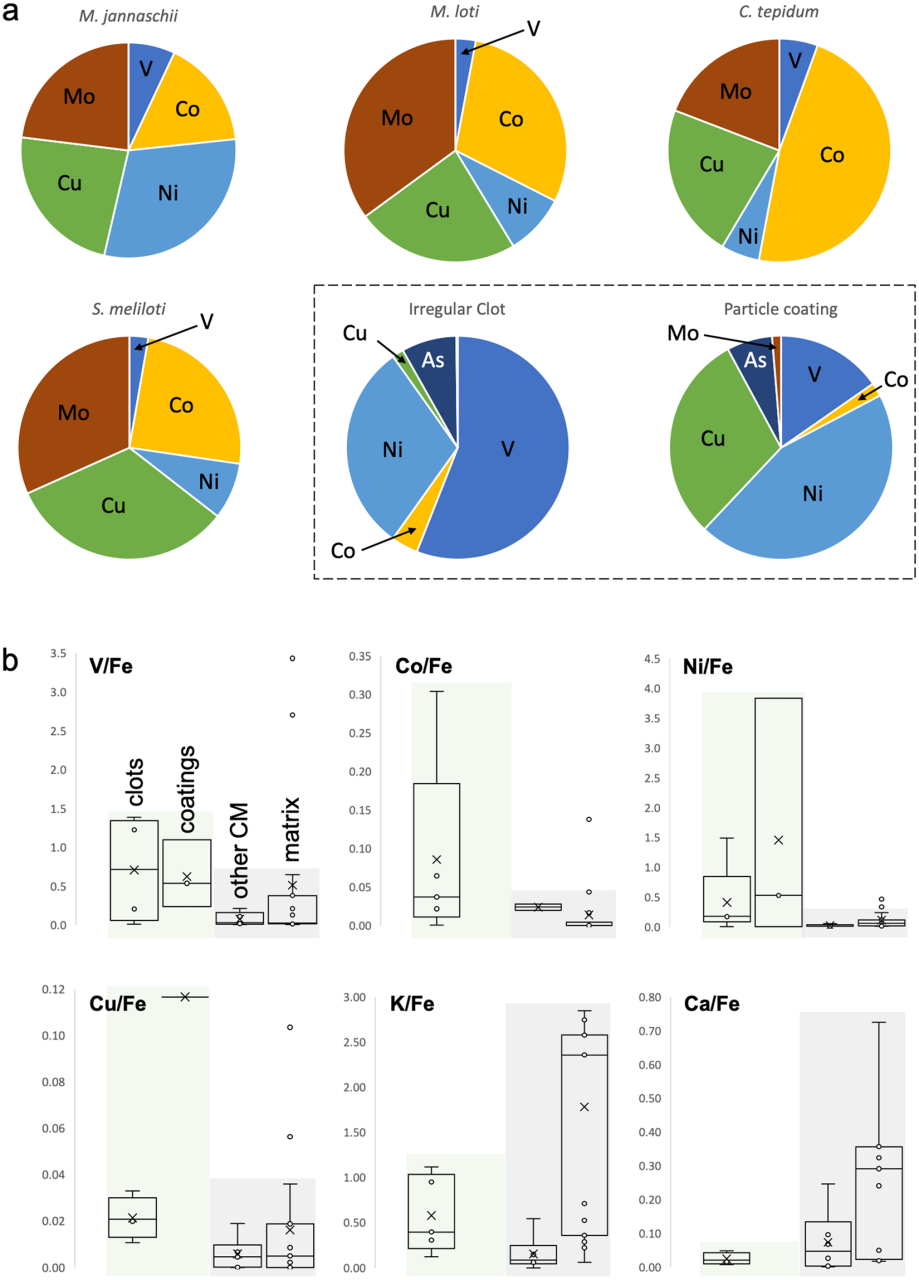
Systematic approach to calculating the palaeo-metallome with reference to modern organisms. (**a**) Comparison between fractional contributions of elements of interest in Josefsdal Chert carbonaceous material and reported prokaryotic metallomes (e.g. Zerkle *et al.*^41^). A metallomic contribution of V, Co, Ni, Cu and As (with bio-essential Fe, not shown) is frequently seen in irregular clots and particle coating morphologies. The least deviating pattern is seen between that of *M. jannaschii* and Josefadal Chert carbonaceous material: V, Ni, Co, Cu and Fe, together with reduced (or absent) requirements of Mn and Mo. The higher dependency on Cu seen for *S. tepidum* and *S. meliloti* more closely aligns its intracellular metal abundances with carbonaceous material in particle coatings, but we suggest that Cu may not be integral to the Josefsdal Chert carbonaceous material, but instead be the result of non-specific uptake due to extracellular polymer-driven resistance to toxicity (after Hickman-Lewis *et al.*^33^). In most cases, the concentrations in putative biomass is lower than that for *in vivo* cells. This is consistent with degradation of biomass during the decay of cellular components. (**b**) Metal:metal ratios for the studied microstructures against values in the matrix demonstrating statistically distinct enrichments of V, Co, Ni, Cu, K and Ca. Enrichments in transition metals and depletions in Ca and K are consistent with bio-accumulation (i.e., in molecular nanomachines) and toxicity mitigation (i.e., against salt stress). The overall differences between significant metal enrichments in clots and coatings (highlighted in green) with respect to flakes and the matrix (highlighted in grey) implies that the accumulation of transition metals in flakes occurs by a different, less efficient process. We propose bio-accumulation for the effective accumulation of metals in clots and coatings, but passive, non-specific accumulation in the case of flakes. Passive accumulation is consistent with the similarity in metal:metal ratios in flakes and the matrix. Where no box plot is shown, either one or both of the elements in question were not present in the region of interest.

Spatially correlated enrichments in trace elements, particularly transition metals, are found throughout the CM of irregular clots and particle coatings (Fig. 6a), but not in generic carbon flakes, where enrichments are localised within microminerals (Fig. S16). The average metal and metalloid enrichments within clotted and coating CM deviate notably from those in flakes and in the matrix, as evidenced by metal:metal ratios for V, Co, Ni and Cu against ubiquitously present bio-essential Fe (Fig. 6b; and compare average enrichments in Table S2 with those in Table S3). Since elemental enrichments in clots and coatings are not due to mineral phases, the diffuse trace element signature in both of these CM morphologies is primary and indigenous to CM. Moreover, since high-resolution optical microscopy was used to ensure that regions of interest were selected far from secondary veins or obvious deformational features (Fig. S9) through which later migration of fluids could have influenced this CM, and since hydrothermal silicification is extremely rapid (initiated in hours^24,25,43^) and provides a ‘time capsule’ of crystallographic preservation, post-diagenetic mobilisation of metals into or out of biological organic matter can be discounted. Furthermore, the rapid sealing of functional groups by silica during earliest diagenesis^25^ inhibits the uptake of metals from the silicifying fluid. Thus, since the CM is highly likely of biological origin (see Supplementary Discussion), we interpret trace element concentrations within this CM as reflecting the *palaeo-metallome* of the precursor biomass.

Caution is yet required in the interpretation of this signature, since it may reflect a combination of original cellular chemical selection^19,61^, very limited chelation of elements during diagenesis^24,25^ and the uptake of elements into extracellular polymers as a response to toxicity^33^. Indeed, cellular and secreted biological molecules are commonly rich in carboxylate groups, peptidoglycan, teichuronic acid and other heteropolymeric polysaccharides, and are likely to retain metals accumulated during life even after death^24,25,62^. An understanding of the biogeochemistry of each element is thus required in order to decode the palaeo-metallome: a challenging, but potentially powerful, bio-indicator.

## Origin of the Palaeo-Metallomic Biosignature

Metals are essential to all life and have widespread utility in molecular nanomachines^19–21,23^. Of the genome, proteome and metallome, the metallome is the only likely candidate for preservation throughout deep time. Indeed, biomarkers indicative of the proteome are not thought to preserve into Archaean sediments^42^. The spatially discontinuous metal-rich signatures observed in our analyses (Figs. 4–5) are a function of micrometric discontinuity in CM, which occurs as dispersed, interleaved fragments between silica crystals (Fig. 2g,h). This distribution is consistent with that of originally cellular material in similarly ancient and younger Proterozoic rocks^59,63^, i.e., micro-scale cross-sectional morphology bears little significance on the biological origin of CM. Generally, µPIXE mapped higher concentrations of elements in regions of visually elevated CM concentrations, however, the patterns of distribution of metals are frequently heterogeneous (Fig. 4c), which may reflect multiple micro-scale sources, i.e., precursor molecular diversity. Although some elements were present below the theoretical detection limit of PIXE (~100 ppm for most elements), the ability to map their presence in discrete energy channels is consistent with their occurrence at low concentrations^56^.

In hydrothermally influenced environments and potentially the wider Archaean ocean, soluble Fe^II^, Ni^II^, Co^II^ and Mn^II^ were more bio-available, whereas insoluble Cu^II^ and Zn^II^ and Mo^IV^ were less so^61,64^. The concentrations of these elements at the ocean-sediment interface would have been elevated by their incorporation into fluids permeating mafic oceanic bedrock. Our measured concentrations corroborate this: in the ambient environment, Cu, Zn and Mo are present in significantly lower concentrations than Fe, Ni and Co. Mo, Cu and Zn were, indeed, seemingly later (syn-GOE) additions to the metallome^61,65^, their importance to biology significantly post-dating the deposition of these rocks. Phylogenomic analyses of Archaean-representative prokaryotes suggest that their metal requirements were consistent with a ferro-sulphidic environment, for instance the necessity for Co^66,67^ and a lacking requirement of Zn^64^, high Cu sensitivity and low metal tolerance in the absence of ferro-sulphidic conditions^20,64^. These expectations are also supported by our results; indeed, almost all expected metallic elements are enriched in both CM and the matrix relative to their concentrations in the modern oceans. The Archaean CM studied further concentrates only a specific range of trace elements relevant to prokaryotic metallomes, including Fe, V, Co, Ni and As^29,30,41,61^, sustaining our hypothesis that clear and recurrent patterns of elemental enrichment in CM reflect the exceptionally preserved metallomic complement of biomass.

These enrichments are further consistent with coordination chemistry involving metalloproteins yielding ion enrichments within biomass^23,68^. Hard and soft Lewis acid and base (HSAB) theory predicts that hard Lewis acids (K, Ti, Fe, V, Cr, Mo, Mn) and soft Lewis acids (Ni, Co, Cu), together with hard Lewis bases, e.g., P, associated with PO_4_^3−^ and H_2_PO^4−^, and S, a hard base in SO_4_^2−^ and soft base in ROH, R_2_S and S_2_O_3_^2−^, would be associated with ligands including H_2_O, ROH, RCO^2−^, CO_3_^2−^, NO_3_^−^, PO_4_^3−^, SO_4_^2−^ and NH_3_, which complex readily in hydrothermal effluent to make elements bioavailable (although SO_4_^2−^ and NO_3_^−^ are generally present at low concentrations in hydrothermal fluids). Coordination complexes formed from hard acids require hard bases. The presence of either carboxylate groups or phosphates is thus useful, e.g. cell wall proteins and EPS in biomass^69^. Moreover, microbial matter is generally extremely effective in the role of metal accumulation based on the fact that biomass (particularly membrane-forming polymers) has an exceptionally high surface area to volume ratio for interaction with metal ions in the immediate environment coupled with a net negative charge due to a preponderance of carboxylate and other lipopolysaccharides, together with peptidoglycan. In both Gram-positive and Gram-negative microbes, this yields an unmatched ability to accumulate metal ions^24^, which have been experimentally demonstrated to be retained after death^25^. The sealing of surficial functional groups by silica during diagenesis^24,25^ also inhibits metal uptake from the silicifying fluid. Coordination with aliphatic compounds or highly cyclised abiotic carbon produced by Fischer Tropsch-type and other wet hydrothermal reactions is, from the point of view of complexation, distinctly less favourable^66^. CM enriched in both hard and soft metals is thus consistent with biologically driven accumulation. This is further consistent with the lack of diffuse elemental enrichments within the negative control – generic CM particles – which instead simply sequester elements in matrix concentrations (Fig. 6b).

Using metal: metal ratios (V/Fe, Co/Fe, Ni/Fe and Cu/Fe, K/Fe and Ca/Fe), we find that strikingly distinct trends emerge between the ratios in clots and coatings when compared with the ratios in flakes and the matrix (Fig. 6b). Clots and coatings generally show higher ratios of V/Fe, Co/Fe and Ni/Fe (as well as Cu/Fe, but see below) that suggest specific directional enrichment reflecting a controlled environment (cf.^22^). Flakes have metal:metal ratios closer to matrix values, suggesting a predominance of passive accumulation. Furthermore, the lower K/Fe and Ca/Fe ratios in clots and coatings than in flakes and the matrix further distinguish these two groups of microstructures. These recurrent patterns of metal incorporation argue for selective incorporation, occurring before post-mortem diagenesis. Diverse spatial concentrations of elements on the micron-scale also suggest slightly different concentration methods in the biogeochemical cycle of each element, whether for metabolism or detoxification.

We thus find that the elemental enrichments reported result from syngenetic environmental-organismal effects (i.e., metallomic compositions related to biomolecules), since diagenetic and taphonomic histories related to secondary alteration can be excluded. CM in both clots and coatings likely consists of intermixed cellular material reflecting a metabolic network or biocoenosis^14,31^, together with extracellular biomolecules and metal-chelating proteins, both of which contain anions (cell proteins and aspartic and glutamic acids) able to chelate metal cations, for example Cu^33,69^. Significant metal accumulation associated with potential siderophores and other highly chelating proteins^70^ not of direct relevance to the metallome would have been removed with minerals (i.e., point sources of extreme enrichment; Figs. 2e,f, S4–S8) during spatial quantification (see methods described in Supplementary Material) and would not hinder the distinction of metal accumulation within CM itself. As noted by Heim *et al*.^58^ it is difficult, and indeed often impossible, to distinguish microbially and abiotically mediated metal accumulation in modern deposits. Consequently, we advocate that inferences of metal accumulation ascribed to microbial activity should only be made based on elemental signatures in CM itself.

## Metabolism of the Precursor Biomass

In the absence of cellular preservation, Linnaean classification is impossible, thus the elemental composition must be conservatively interpreted to represent a community of organisms. This makes comparison with previously reported single organism metallomes (e.g.^29,30,41^) challenging, and suggests that a more appropriate comparison would be with metabolism-specific metallomes^30,41^. δ^13^C values of this kerogen are consistent with multiple metabolisms known or hypothesised to have developed by the Palaeoarchaean, including anaerobic photosynthesis, sulphur and sulphate reduction, nitrogen fixation and methanogenesis (see Fig. 3), each of which may have existed in this microbial biome. Although many metabolisms depend upon a range of enzymatic co-factors^23^, certain metabolisms show specific requirements: hyperthermophile methanogens such as *Methanocaldococcus jannaschii* exhibit elevated metallomic fractional requirements of Ni, Co, Cu and V relative to other metabolisms, whereas Zn and Mn are required at substantially reduced concentrations^22,40^. Until Mo became more bioavailable at 2.5 Ga, nitrogen fixation is thought to have relied upon Fe and V^71^. Nonetheless, off-axis hydrothermal systems are known to mobilise small quantities of Mo^72^ and the anoxic weathering of volcanics could have sustained nitrogen fixation using Mo-nitrogenase at even 3.2 Ga^73^. Consequently, the lack of detection of appreciable concentrations of Mo in JC CM may simply reflect an absence of Mo-based nitrogen cycling in this environmental setting. Differential requirements for elements, although complex and highly dependent upon their palaeoenvironmental context, could conceivably define a biosignature for contributions by specific metabolisms in the precursor biomass and explain, as an example, the high concentrations of V retained within the studied kerogen. In Fig. 6a, we report µPIXE-determined spatially quantified elemental concentrations as percentage contributions to metallomes following the approach of Zerkle *et al*.^41^. Strong similarities with the fractional requirements of certain trace elements in specific metabolic machinery emerge.

Fe, Na, Mg, P, S, K and Ca are bio-essential^41,61,65,74^ and, with the exception of Ca, are distributed throughout the clotted and coating-like CM studied herein. This exceptional elemental complement alone is strong evidence for this CM having a biogenic origin. V is considered either bio-essential or bio-functional^40,70^ and has high retention potential within CM^62^. Very high fractional contributions of V in clots and coatings may stem from this preferential retention or original enrichment and, while potentially an overestimate relative to enrichments in other elements, suggests metabolic contributions from methanotrophic and diazotrophic organisms^23,41^. V is essentially absent in the matrix; this can be explained by its concentration within CM which, while abundant in the regions of interest, is negligible in bulk sample (<10 ppm; Fig. S11). Cu appears to have been incorporated into the metallome only from the Mesoarchaean onward^61,65^, thus its enrichment in CM probably reflects non-specific sequestration by biomolecules due to toxicity, as observed in polysaccharides associated with modern microbes^33^. Indeed, the elevated Cu/Fe ratios observed in the studied CM microstructures may be explicable by passive absorption by EPS^22,33^. We do not suggest that Cu was integral to the metabolic network of this 3.33 Ga microbial community, since this is incompatible with most theories of evolutionary biology, and since REE+Y palaeoenvironmental reconstruction (Fig. 2a) does not suggest any aspect of an oxygen oasis in the JC. Co, Ni and As are considered either bio-essential or bio-functional and are enriched within individual sulphide grains and more diffusely within CM. The modelled metallomes of chemoautotrophic methanogens and, more broadly, (hyper)thermophiles, demonstrate an enrichment of Ni to a fractional contribution twice that of other metabolisms^30,41^. Co and Ni are furthermore important components of the metallomes of methane- and nitrogen-cycling chemolithotrophs^75^, which are plausible metabolic precursors in light of the petographic context of this carbonaceous material: lithotrophs would be most likely associated with particles^31^. Such primitive, anaerobic metabolisms^35^ are also highly compatible with the thermophilic hydrothermal biomes evidenced by our trace and REE + Y environmental reconstruction (Figs. 2a, S10–S11) and thus the measured pattern of enriched Fe, Ni, Co, V and As is concordant with the expected metabolic network in this hydrothermal-lithotrophic palaeoenvironment. Of these elements, As is the most challenging case to evaluate since, although As-based metabolisms have been inferred at 2.7 Ga^22^, As is not widely used in metalloenzymes. We propose that its accumulation in these CM microstructures may either result from biological use or non-specific detoxification uptake as is potentially the case for Cu (e.g.^33^). Other bio-functional elements, including Ba, W, Cd and Sn, were not detected during our analyses. These elements are either absent from the palaeo-metallome of any biomass analysed or present in vanishingly small concentrations below the limit of detection. This discussion highlights the importance of evaluating a putative palaeo-metallome in light of the biogeochemistry of each of its constituent elements.

In conclusion, a thermophilic, hydrothermally influenced biome in the Palaeoarchaean would demand a metallome consisting of bio-essential elements together with metabolism-specific V, Cr, Co, Ni, As and W, and possibly others^61,74^. Due to bio-availability constraints in anoxic environments, Zn, Cu and Mo likely played a minor metallomic role on the Earth prior to the GOE^23,61^. Internally heat-driven, anaerobic conditions were plausibly more clement to thermophiles demanding metallomic V, Co, Ni, As and W, which is sustained by trophic-level comparisons with reported metallomes^29,30,41^. Our results are further consistent with expected elevated ion concentrations in the Archaean oceans^21,64^. We suggest that the trace element enrichments characterising both irregular clots and particle coatings in the 3.33 Ga Josefsdal Chert represent bioaccumulation (after^27,28,68^) in a hydrothermally influenced shallow-water biome. The trace element signature of this CM carries a strong resemblance to anaerobic, methanogenic or diazotrophic, thermophiles (and likely a combination of the two, and potentially other metabolisms such as sulphate reduction) in terms of both absolute and relative enrichments and fractional contributions to the palaeo-metallome (Fig. 6a). Moreover, metal:metal ratios show clear distinctions between the elemental compositions of irregular clots and particle coatings *versus* flakes and the matrix. The differences in these ratios are incompatible with passive accumulation, since all CM morphologies were deposited contemporaneously. *In situ* carbon isotope fractionations corroborate our biological interpretation of clots and coatings: values centred around values less negative than −20‰ are consistent with fractionation by nitrogen-cycling, sulphur-cycling, methanogenic and/or anoxygenic phototrophic microbes, although slightly heavy to infer methanotrophy^9,15–17,76,77^. We therefore propose that clotted and coating morphologies of CM are relics of an early Earth chemosynthetic (potentially chemolithotrophic) ecosystem.

The wide range of isotope fractionation in flakes, encompassing both positive and negative values, does not resemble known biological distributions^16,17^, but does exhibit a certain similarity to hydrothermal carbonaceous products^53^. Whether this indicates that the flakes and other generic CM analysed derive from abiotic sources alone is difficult to assess from solely carbon isotopes, significant though such a statement would be in such ancient material. Answering such a question would likely require high-resolution structural and geochemical analyses on micro-sampled material from multiple CM morphologies (see procedures described in^78^). Consequently, this dataset alone implies that these flakes resulted from diverse, non-specific processes.

## Implications for the Metabolic Networks of Early Ecosystems

Elements must be bio-available in their ambient chemical environment to become an integral part of the metallome. These results provide the first estimation of the trace element budget of Archaean biomes (see also^79,80^), and support that Palaeoarchaean hydrothermally influenced habitats were rich in many of the elements required by the thermophilic prokaryotic metallome. Not all bio-functional metallic elements are present (e.g. W and Sn), and thus the metallome may have undergone degradation as a function of cell death and taphonomy, but it is more likely that these elements simply played no role in metabolisms of the precursor biomass since preservation in fossiliferous chert *Lagerstätten* is otherwise exceptional^5,9,37,38^. Palaeo-metallomic biosignatures are likely a function of both original cellular concentrations and the preservation potential of the intra- and extra-cellular components with which the element was associated (i.e., metalloproteins *versus* cytoplasm *versus* EPS). In Archaean sediments, the palaeo-metallome is most accurately assessed as a community signal. The approach pioneered herein opens new avenues of life detection in geological materials of any age and an opportunity to re-interrogate contentious putative biosignatures. Most significantly, it provides a means of investigating biogenicity in the absence of cellular preservation. In the near future, the co-occurrence of trace elements in possible CM will be measurable during Mars rover missions, and this type of biosignature could be observed with the PIXL instrument aboard the Mars 2020 rover^81^. Since Mars rovers lack the microscopy capabilities to observe individual microfossils, elemental signatures associated with putative biomass should be considered highly strategic biosignature targets.

In terms of the early terrestrial biosphere, our findings empirically support that the hypothesis of Frausto da Silva and Williams^20^ – that modern biological dependency on trace elements was influenced by the environments in which early life evolved – is correct. On a regionally thermophile early Earth, hydrothermally influenced oceans were environments relevant to the evolution of the prokaryotic metallome. Since the early aeons accounted for a large portion of microbial evolutionary history^3,37,82^, it is perhaps unsurprising that these elemental legacies have endured.

## Methods

### Sample collection

Samples were collected during field campaigns to the Barberton greenstone belt between 1999 and 2014, during which time a detailed appraisal of the stratigraphy and sedimentology has been conducted^5,31,32,38^. The studied samples were taken from the Josefsdal Chert between Ekulindeni, Mpumalanga province, South Africa, and Bulembu, Hhohho region, Eswatini. Three major sets of outcrops, from which the samples studied herein were obtained, are located at: 25°57.945′N, 31°04.720′E; 25°57.945′N, 31°04.798′E; 25°57.941′N, 31°04.722′E.

### Fundamental petrographic characterisation

For optical microscopy, an Olympus BX51 microscope (CNRS-CBM, Orléans) was used. Unless stated otherwise, all optical images are in plane polarised light. Raman spectroscopy used a WITec Alpha500 RA (CBM, Orléans) equipped with a green laser at 532 nm wavelength and a laser power of 5 mW to avoid thermal alteration of the sample. Raman geothermometry used the carbonaceous materials-based geothermometer of Kouketsu *et al*.^54^. Qualitative identification of spectral characteristics suggested our materials reflect medium metamorphic grade (i.e., lower greenschist facies), after which we used Equation 2 of^54^) as follows: T(°C) =−6.78(FWHM–D2) + 535, where FWHM is the full width half maximum and D2 is a band in the first-order disordered region (i.e., ~1200–1700 cm^−1^). SEM analyses used Hitachi T3000 (ITSO, Orléans) and JEOL (Dipartimento BiGeA, Università of Bologna) instruments operating at 5 keV and 15 keV. Elemental maps were run for 150–200 frames in order to accrue sufficient counts, and point analyses for 100–300 s for the same reason.

### Particle-induced X-ray emission spectroscopy

Particle-induced X-ray emission (µPIXE) combined with Rutherford Backscattering Spectrometry (RBS) was performed using the microbeam beamline of the AIFIRA facility (CENBG, Gradignan), which is described in detail in Sorieul *et al*.^55^. This used the 3 MeV proton microbeam (1 µm) with a current of 200 pA, and a high energy range of 40 keV to mitigate the problem of overlap amongst K and L X-ray lines^57^. Three Si-detectors (one RBS and two PIXE) collected data: the RBS was used for charge monitoring, as lithological samples are insulators, whereas the two PIXE detectors were used for elemental quantification and mapping. The first PIXE detector was equipped with an Al-“Funny Filter” (thickness 100 µm, hole size 2 mm) and a Kapton filter (thickness 50 µm). Kapton is used to filter out elements at lower atomic weight, and is therefore key to determining the true concentrations of heavier trace elements, particularly for those of Ti and higher atomic mass. Greater hole size increases count rate, but has no effect on the rate of individual element detection. The second PIXE detector was equipped with an Al-“Funny Filter” (thickness 100 µm, hole size 1 mm) and was used for complete characterisation of the sample. No filter was used for the RBS detector. Each analysis was allowed to run for 8–12 hours. All analyses were conducted with a dead time of less than 10% in order to avoid pile-up and spectra distortion. Dependent upon the size of the region of interest, scans are either 50 µm, 100 µm or 200 µm squares. PIXE is a non-destructive, non-invasive, *in situ* method for measuring trace element data in cherts when compared with efforts involving HF total digestion. Multiple examples of each morphology of carbonaceous matter have been measured to produce a statistically significant dataset, depending upon their abundance in the studied samples. The matrix invariably contains a small amount of disaggregated carbonaceous matter of indeterminate origin, thus serves as the ideal benchmark against which elemental concentrations of specific larger microstructures are normalised (i.e. ambient conditions). For each sample, multiple analyses of the matrix adjacent to microstructures of interest were conducted, providing the average matrix value against which analyses of carbonaceous material within that microstructure were normalised. X-ray counts can be converted into concentrations following the rationale and methods described in Halden *et al*.^56^ and Campbell *et al*.^57^ which detail the utility and capability of PIXE of determining spatially resolved concentrations of the elements of interest at micrometric resolutions.

The quantification of PIXE data involves a sequential workflow using three programs: SupaVisio, SIMNRA and Gupix^57,83,84^. SupaVisio permits the selection of regions of interest, the spectra of which are extracted from the spectrum of the entire region of analysis. Each extracted RBS spectrum was treated with SIMNRA for charge determination^84^. The charges determined were used for the quantification of sixteen elements of interest (P, S, K, Ca, Ti, V, Cr, Mn, Fe, Co, Ni, Cu, Zn, As, Zr, Mo) with Gupix^83^. The algorithm used by Gupix^83^ calculates a limit of detection for the alpha- and beta-ray (K) of each element (quantification of L and M rays is also possible, though was not of relevance to the elements in question), from which the user assesses the presence or absence of the element. Where close to the limit of detection (within 10% considering analytical error), we took a conservative approach and deemed the element absent. Therefore, all stated concentrations are low estimates. In all quantifications, we consider a ‘mineral effect’. This was identified based upon the element maps generated in SupaVisio, since while many trace elements are broadly enriched within carbonaceous matter, they are occasionally especially enriched within small regions (i.e. minerals). Discounting any quantified analysis in which the value determined is more than five times the average of all other analyses of that type removes the ‘mineral effect’ for that element. These regions were often found to correlate with optically identifiable mineral phases. The application of the ‘mineral effect’ may prevent the quantification of concentration within an element at depth where it is not directly identifiable by petrographic observation but nonetheless skews the results to higher averages (pre-screening of the regions of interest was conducted using high-resolution optical microscopy before µPIXE). This has been done on an element-by-element basis such that only one datapoint will be removed from the overall quantification, thus maintaining the integrity of the statistic.

There is no direct correlation between the appearance of concentrations in the maps, which are a function of the number of counts at any specific point, and the concentration (ppm) of that element, which is a function of the detector-specific quantification of that element according to the algorithm of Gupix (see^56,57,83^). Although the detection of some elements is below the theoretical detection limit of PIXE (~100 ppm for most elements), the ability to map their occurrence in discrete energy channels is consistent with their occurrence as shown in the individual element maps, but at concentrations well below the theoretical limit of detection for PIXE (<100 ppm)^56,57^. The limit of detection (LOD) is defined as the concentration of an element that would give rise to an X-ray peak having an intensity equal to the three-sigma fluctuation of the background underlying the peak. In GUPIX software, LOD is defined as 3 times the square root of the background over 1 full-width half-maximum centred on the principal peak centroid. This corresponds to the LOD definition used for fluorescence spectrometry. The value of LOD can vary with the concentration of the detected element, the preparation of the sample and more generally with the conditions of analysis. In order to achieve both the best conditions of analysis and the lowest background, we used the soft borosilicate glass NIST1411 standard as a reference material. We also prepared the samples accordingly to avoid any parasitic radiation due to the interaction of the beam with the chert matrix. PIXE standardisation is based on the accurate determination of an instrumental constant independent of the X-ray energy. In practice, several uncertainties arose mainly from the inaccurate characterisation by the detector and uncertainties in X-ray transmission. GUPIX software takes into consideration all of these deviations from the ideal measurement through the determination of a sole parameter termed the “H-factor”. For that purpose, two standards were used: soft borosilicate glass NIST1411 and stainless steel AISI 15-7PH. The combination of these two standards covers the energy range from 1 to 20 keV in which all the potential trace elements could be detected. NIST1411 was also used to evaluate the matrix effect and finely tune the beam conditions and detection set-up for the analysis of these cherts.

### Carbon isotope ratio mass spectrometry

*In situ* carbon isotope ratio determinations were performed using a CAMECA IMS1280 large-geometry SIMS instrument at the Swedish Museum of Natural History operating in scanning ion imaging mode. Following a 60 sec. pre-sputter with a 20 kV incident energy, ca. 1 nA Cs beam rastered over a 25 ×25 µm area to remove the gold coating, the beam was reduced to a critically focussed <1 µm analytical spot, with a beam current of ca. 100 pA, which was rastered over an area of 20 × 20 µm during acquisition of data. A low energy electron flooding gun was utilised to prevent charge build up on largely insulating target areas. Sputter secondary ions were steered back onto the ion optic axis using the dynamic transfer optical system, a secondary raster that is synchronised with the primary raster to permit acquisition of data from a scanned area at high mass resolution. The secondary ion species ^12^C^-^ and ^13^C^-^ were separated at a mass resolution of 4000 (M/ΔM), in order to resolve ^13^C from ^12^C^1^H and detected sequentially in the axial ion-counting electron multiplier (E_Max_) with counting times of 1 sec. and 5 sec. respectively over 100 cycles. The resulting ion images were processed using CAMECA Winimage2 software to identify regions of interest (ROIs) of high C concentration, from which the 13 C/12 C ratios were calculated, with the 44 ns E_Max_ deadtime correction applied at the pixel level. Analyses of an in-house reference material, Cpyr2, a pyrolised graphite disk with a δ^13^C_PDB_ value of −35.4‰ (G.D. Layne, Memorial University of Newfoundland, pers. comm.) were performed using exactly the same primary beam and raster conditions in order to retain identical sputtering conditions and, hence, identical mass bias relative to the target graphitised carbonaceous material. Nonetheless, the much higher secondary count rates from a 100% graphite target precluded use of the EM and instead data were acquired simultaneously in two low noise Faraday Cups (FCs), ^13^C in the axial FC2 and 12 C in L’2 FC operating at mass resolutions of 4000 and 2530 respectively. The instrumental mass bias determined from the Cpyr2 analyses was then applied to the ratios obtains from the target ROIs to yield their δ^13^C_PDB_. Some of the near-positive and positive values were derived from high isotopic values located at the very edge of the rastered area and could result from a bias in beam centring, which may generate a concomitant bias in isotopic values.

### Inductively coupled plasma mass spectrometry

Fresh rock chips, unaffected by secondary alteration and veining, were selected and ground to a particle size of less than 4 µm for ICP-MS analysis. ICP-MS analyses were conducted at the Centre de Recherches Pétrographiques et Géochimiques (CRPG), Nancy, France, using Thermo Fisher ICap 6500 and Agilent 7700X instruments.

## Supplementary information

Supplementary Information.
