# Supplementary material for: Metallomics in deep time and the influence of ocean chemistry on the metabolic landscapes of Earth’s earliest ecosystems
Source: Sci Rep. 2020 Mar 18;10:4965. doi: 10.1038/s41598-020-61774-w (PMC7080831; doi:10.1038/s41598-020-61774-w)
Supplement: Supplementary file 1 — Supplementary Information. [file 41598_2020_61774_MOESM1_ESM.pdf]

Supplementary Material for: Metallomics in deep time and the influence of ocean chemistry on the metabolic landscapes of Earth's earliest ecosystems

Keyron Hickman-Lewis, Barbara Cavalazzi, Stéphanie Sorieul, Pascale Gautret, Frédéric Foucher, Martin J. Whitehouse, Heejin Jeon, Thomas Georgelin, Charles S. Cockell, Frances Westall

[1]CNRS Centre de Biophysique Moléculaire, Rue Charles Sadron, 45071 Orléans, France
[2]Dipartimento di Scienze Biologiche, Geologiche e Ambientali (BiGeA), Università di Bologna, Via Zamboni 67, I-40126, Bologna, Italy
[3]Department of Geology, University of Johannesburg, PO Box 524, Auckland Park 2006, Johannesburg, South Africa
[4]University of Bordeaux, CNRS, IN2P3, CENBG, 19 Chemin du Solarium, 33175 Gradignan, France
[5]Université d'Orléans, ISTO, UMR 7327, 45071, Orléans, France; CNRS, ISTO, UMR 7327, 45071 Orléans, France; BRGM, ISTO, UMR 7327, BP 36009, 45060 Orléans, France
[6]NORDSIM, Department of Geosciences, Swedish Museum of Natural History, Frescativägen 40, 114 18 Stockholm, Sweden.
[7]Sorbonne Universités, UPMC Paris 06, CNRS UMR 7197, Laboratoire de Réactivité de Surface, 4 Place Jussieu, 75005 Paris, France
[8]UK Centre for Astrobiology, School of Physics and Astronomy, University of Edinburgh, James Clerk Maxwell Building, Edinburgh, EH9 3JZ, United Kingdom

Correspondence: keyron.hickman-lewis@cnrs-orleans.fr

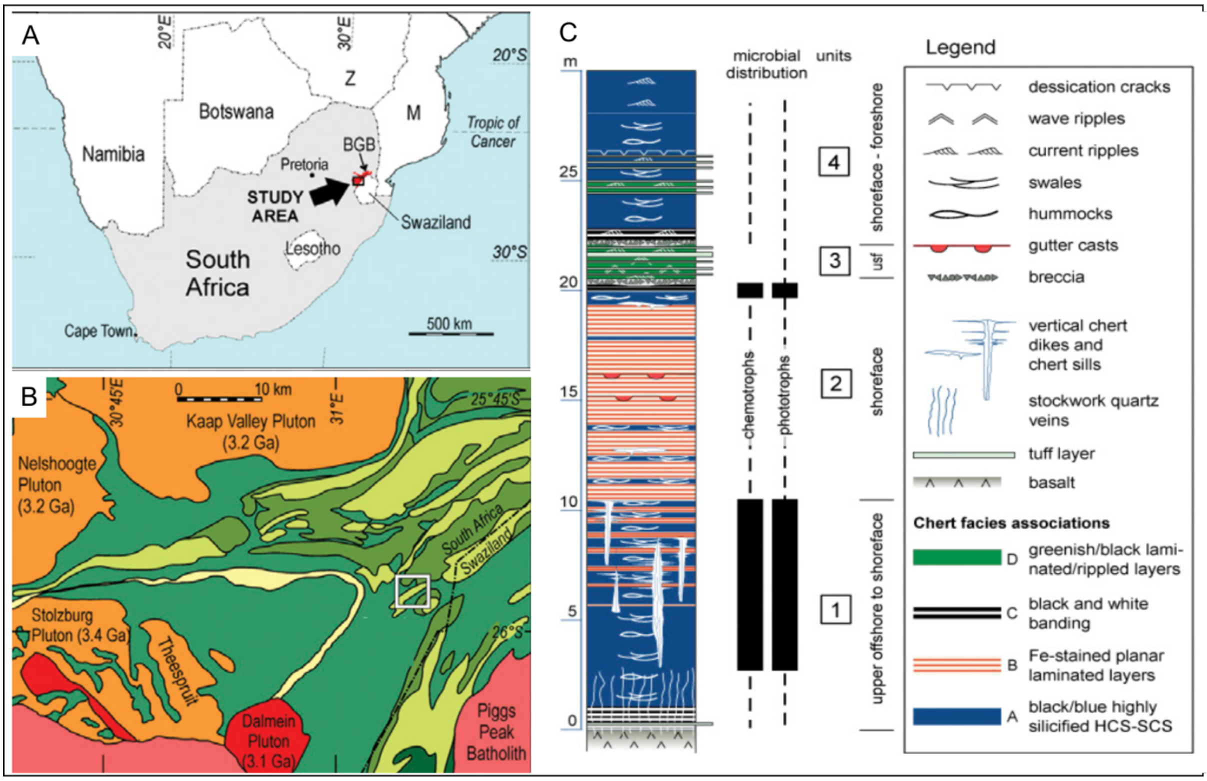

Figure S1. Location, geological setting and stratigraphy of the Josefsdal Chert (equivalent to K3c; Lowe and Byerly, 1999). **A**) Location of the Barberton greenstone belt within South Africa and Swaziland. **B**) Location of the study area (white box) within the Barberton

greenstone belt, close to Msauli and the border with eSwatini. **C**) Stratigraphic column of the Josefsdal Chert; the studied samples come from Facies C, hydrothermally influenced banded black and white cherts from units 3 and 4. Adapted from Westall et al. (2015).

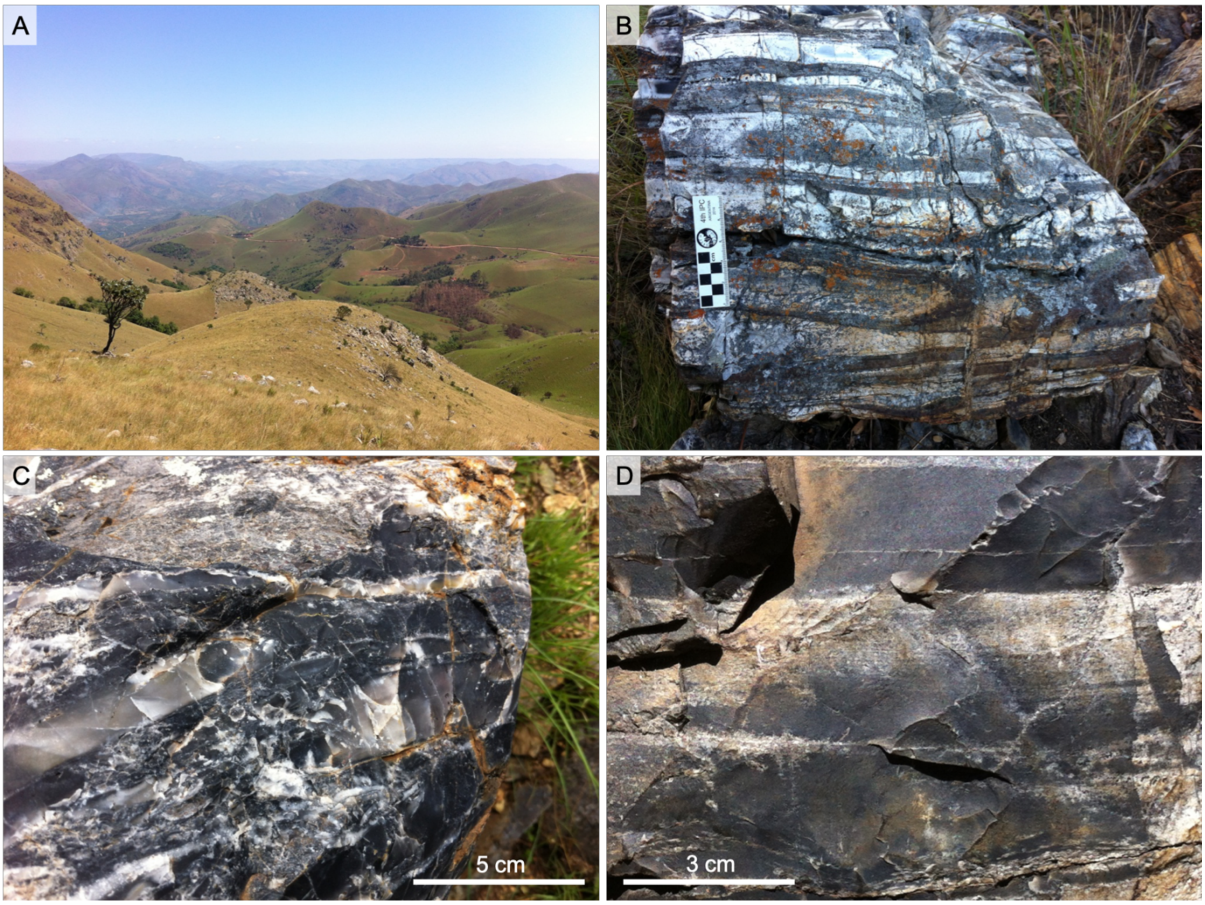

Fig. S2. Selected field photographs from the studied outcrops. **A**) Field photograph of the studied Josefsdal Chert outcrops near Msauli. **B**) Black and white banded chert representative of, e.g., samples 12SA09 and 12SA16 (sample 12SA09 shown). Squares on scale bar = 1cm. **C-D**) Massive black chert with faint laminations characterised by clotted textures in thin section, showing collection localities of samples 99SA07 (C) and 14SA01 (D). Note the spatial delimitation of carbon-rich regions and white silica-rich regions corresponding to elevated silica input. Detailed information regarding these outcrops and the other lithologies of the Josefsdal Chert can be found in Westall et al. (2015).

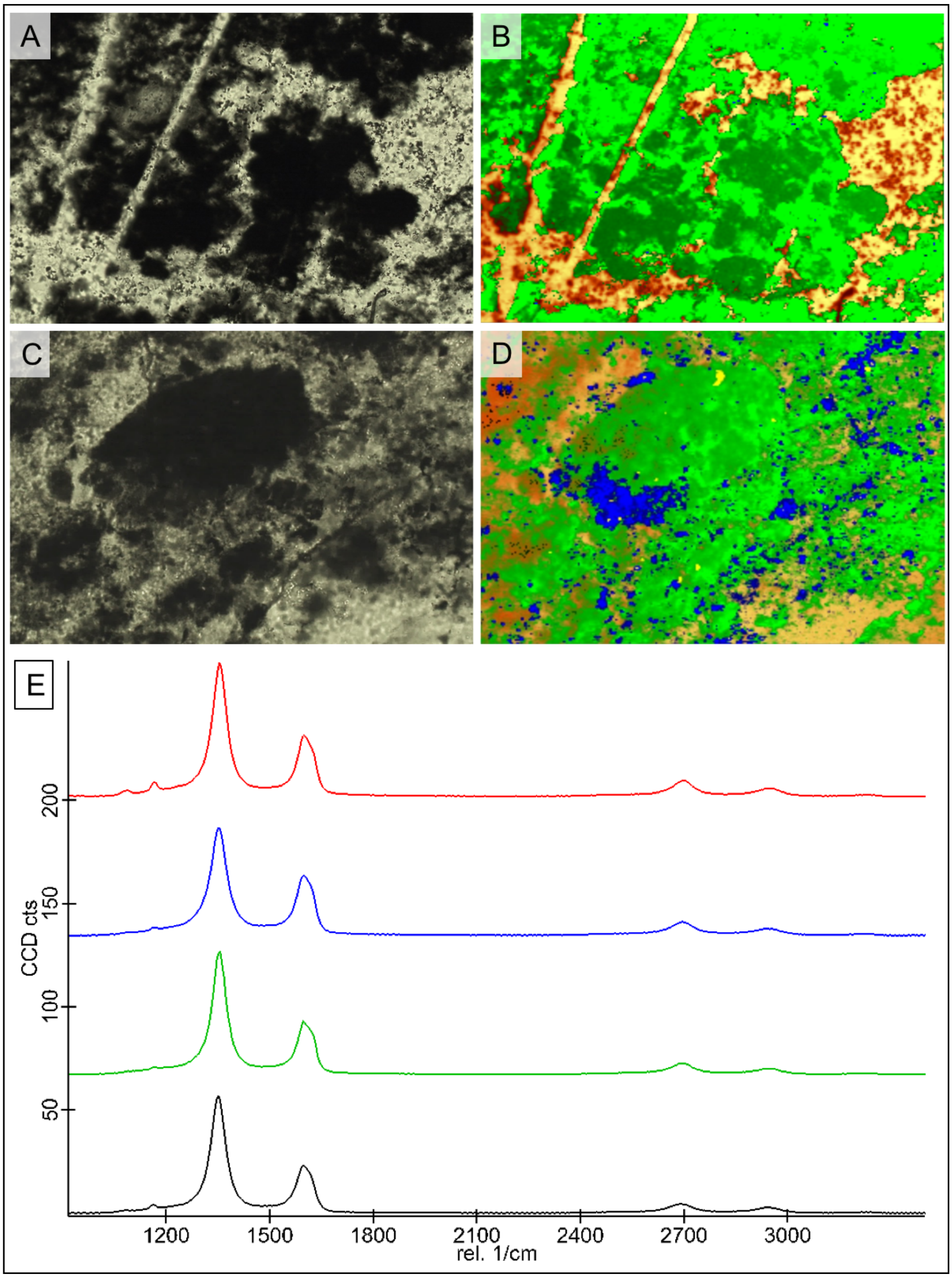

Figure S3. Optical microscopy and Raman spectroscopy mapping for regions of interest. **A-B**) Irregular clot in clotted carbonaceous chert. **C-D**) Volcanic particle coated in carbonaceous material in a volcaniclastic sediment. Green = carbon; yellow-orange = silica and blue = anatase. **E**) Averaged Raman spectra of carbonaceous matter within the structures of interest for four samples (99SA07, 12SA09, 12SA16 and 14SA01).

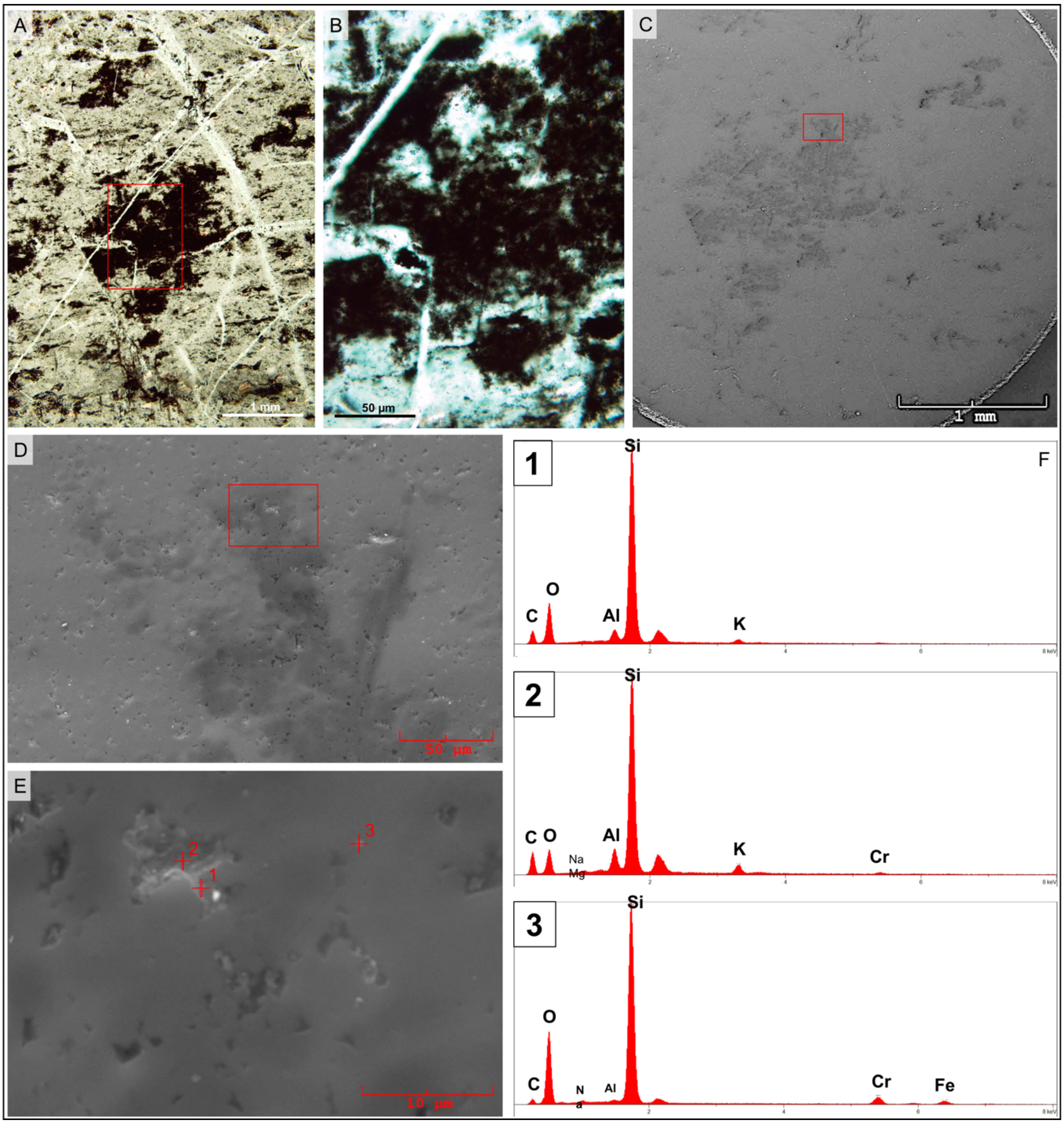

Figure S4. SEM-EDS of irregular clots in clotted carbonaceous chert (sample 99SA07, region 1). **A**) Transmitted light photomicrograph of irregular clot in thin section; boxed area detailed in B. **B**) Detail of irregular clot. **C**) Electron image of the same clot; red box indicates region of D. Carbonaceous matter is dark grey, silica is light grey. **D**) Electron image of limb of clot; red box indicates region of E. **E**) Regions of EDS point analyses (numbered crosses). **F**) EDS point analyses on Au-coated sample accounting for point enrichments in Al, Na, Mg, K, Cr and Fe. Spectra 1 and 2 are aluminous phyllosilicates, whereas spectrum 3 is likely a sub-micron chromite spinel.

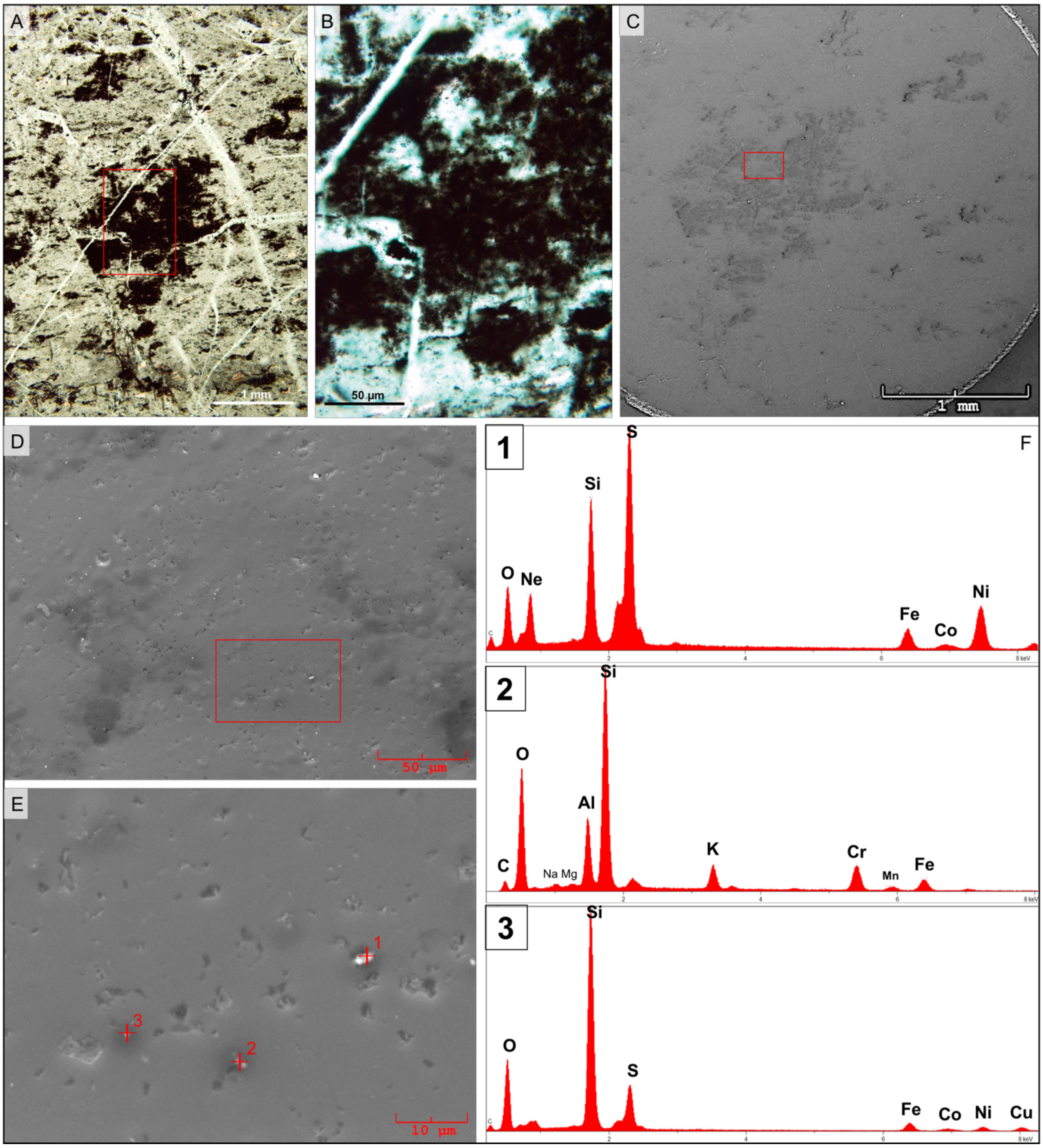

Figure S5. SEM-EDS of irregular clots in clotted carbonaceous chert (sample 99SA07, region 2). **A**) Transmitted light photomicrograph of irregular clot in thin section; boxed area detailed in B. **B**) Detail of irregular clot. **C**) Electron image of the clot in A; red box indicates region of D. Carbonaceous matter is dark grey, silica is light grey. **D**) Electron image of interior of clot; red box indicates region of E. **E**) Regions of EDS point analyses. **F**) EDS point analyses on Au-coated sample accounting for point enrichments in Al, Na, Mg, S, K, Cr, Mn, Co, Fe and Ni. Spectra 1 and 3 are Fe-Ni sulphides (pyrite-pentlandite), whereas spectrum 2 is an aluminous phyllosilicate.

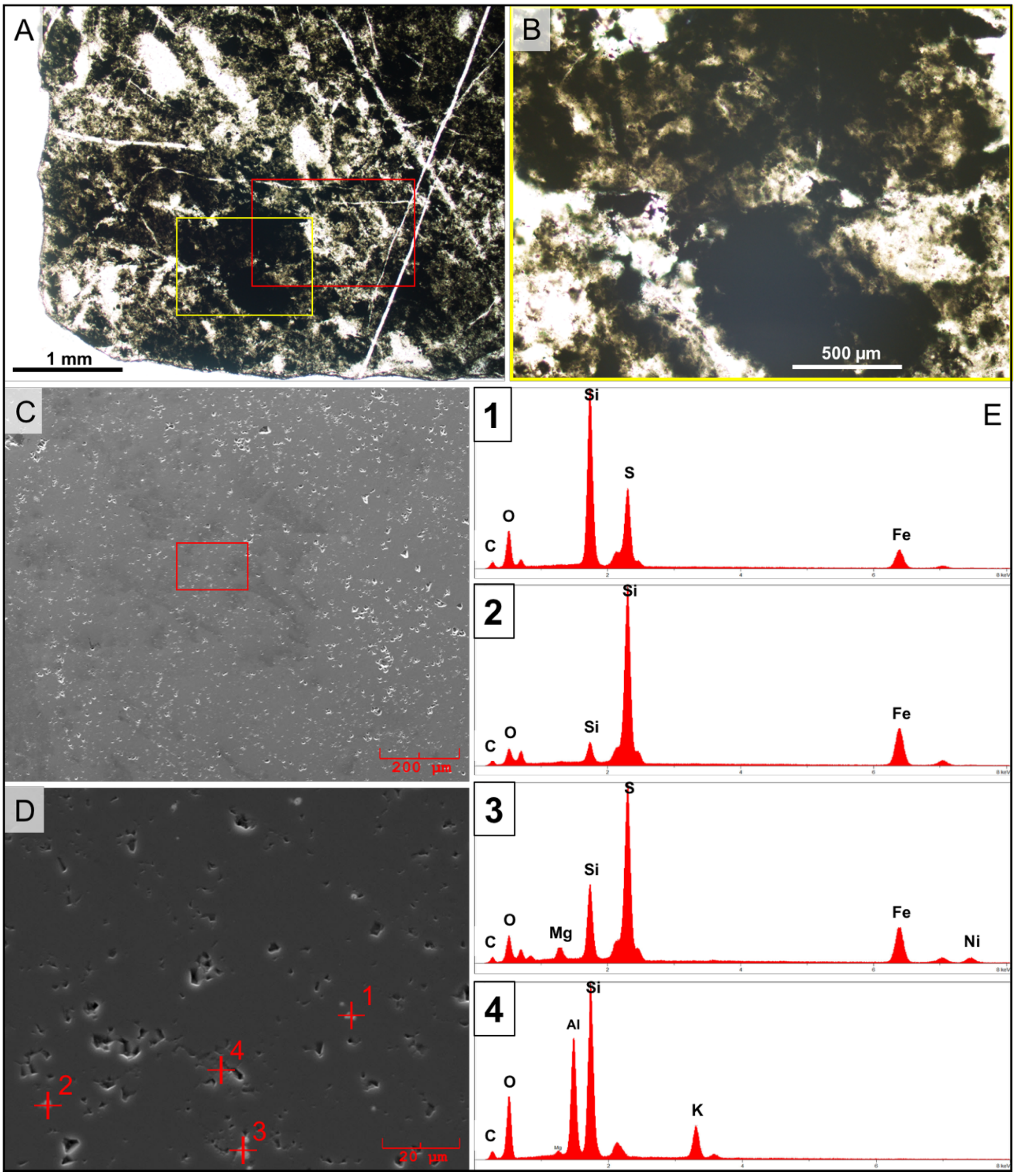

Figure S6. SEM-EDS of irregular clots in clotted carbonaceous chert (sample 12SA09). **A**) Transmitted light photomicrograph of irregular clot in volcanogenic groundmass in thin section. Yellow box indicates region of B; red box indicates region of C. **B**) Detail of irregular clot. **C**) Electron image of the clot in A; red box indicates region of D. Carbonaceous matter is dark grey, silica is light grey. **D**) Region of EDS point analyses. **E**) EDS point analyses on Au-coated sample accounting for point enrichments in Al, Na, Mg, S, K, Cr, Mn, Co, Fe and Ni. Sepctra 1 and 3 are Fe-Ni sulphides (pyrite-pentlandite), whereas spectrum 2 is an aluminous phyllosilicate.

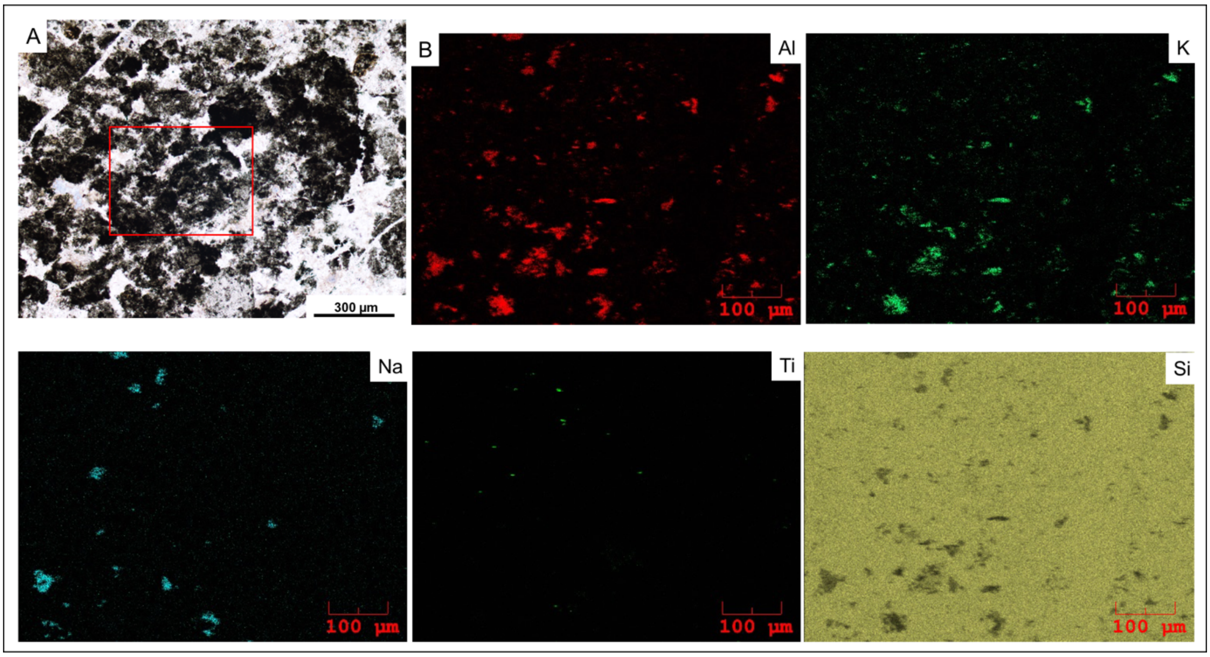

Figure S7. SEM-EDX mapping of carbon-coated volcanic grains (sample 14SA01). **A**) Transmitted light photomicrograph of carbon-coated volcanogenic grains. Red box indicates the region of the EDX maps. **B**) EDX maps of Al, K, Na, Ti and Si. Correlated Al-K-Na-(Mg)-(Fe) indicates particles of mafic and felsic genesis, likely feldspars, pyroxenes and minor olivine, the latter represented by slight enrichments in Mg. Na-Al-K enrichments in tabular particles are probably relict feldspars. Ti is enriched in ilmenite and rutile. Ti corresponds to rutile crystals. SiO_2 is overwhelmingly the major constituent of the rock.

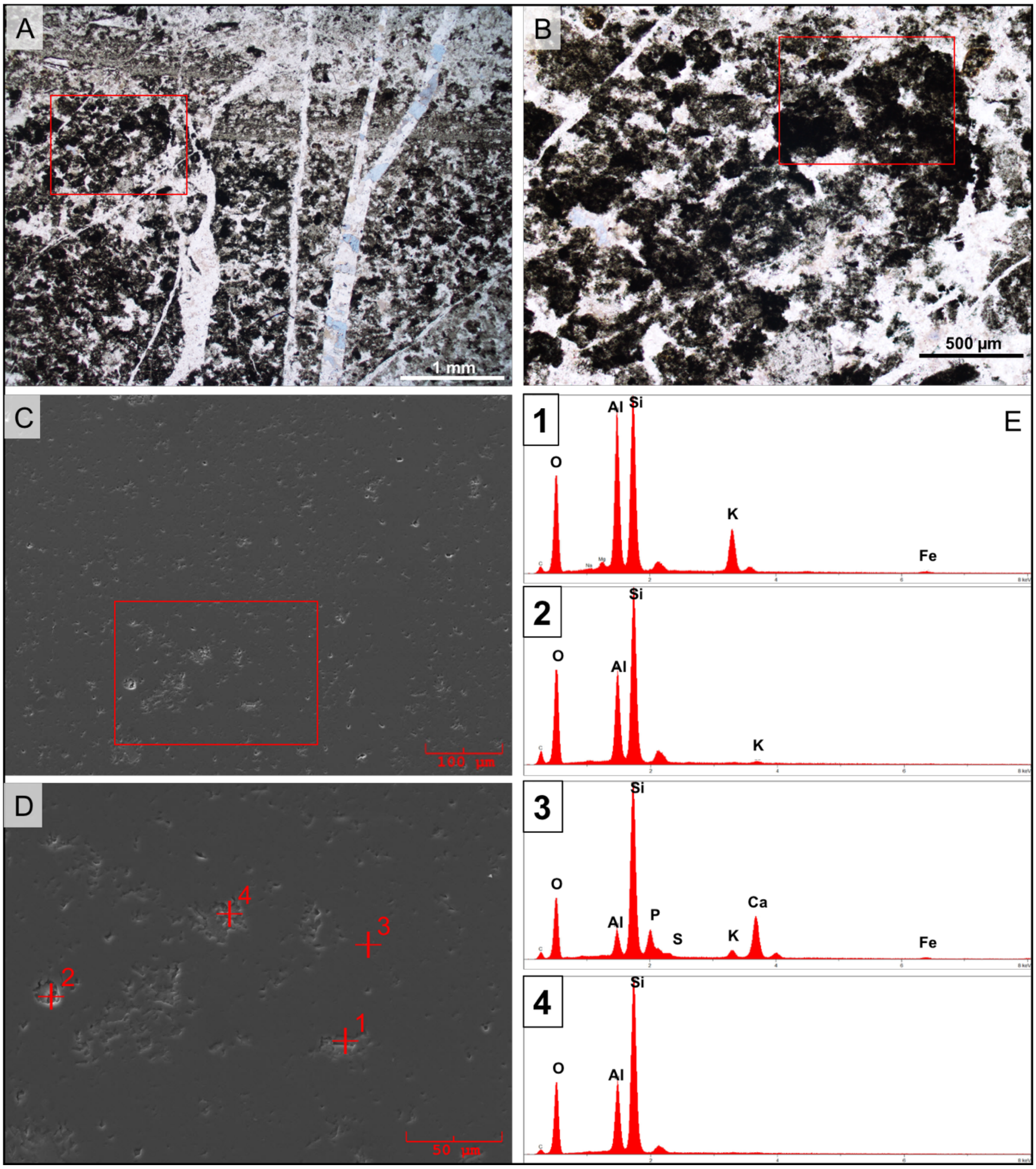

Figure S8. SEM-EDS of carbon-coated volcanic particles (sample 14SA01). **A**) Petrographic image of carbon-coated volcanogenic particles. Red box indicates region of B. **B**) Detail of carbon-coated volcanic particles. Red box indicates region of C. **C**) Electron image of the particle in B; red box indicates region of D. **D**) Region of EDS point analyses. **E**) EDS point analyses accounting for point enrichments in Al, Na, Mg, P, K, Ca and Fe. 1 is an altered alkali feldspar, 2 and 4 are aluminous phyllosilicates, 3 is apatite with pyrite and K-feldspar (both unseen) seemingly within the region of analysis.

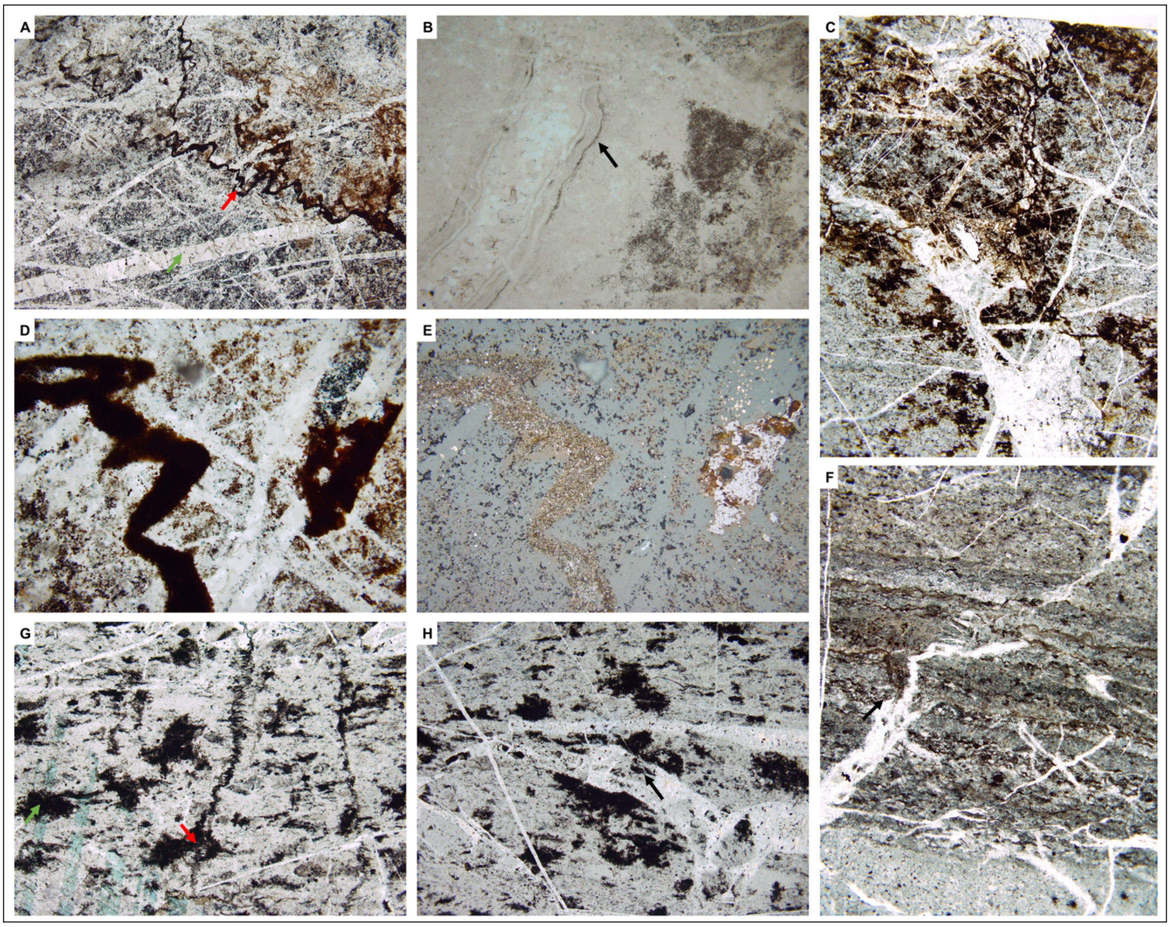

Figure S9. Optical petrographic images indicating features or fabrics of demonstrably secondary origin, and which were avoided in the selection of regions of analysis in order to assure that the recorded geochemical signature is primary. A) Stylolite (red arrow) resulting from pressure solution fronts, and large secondary megaquartz vein (green arrow). B) Botryoidal chert fabrics developing within penecontemporaneous veins and leading to the secondary displacement of carbonaceous material (arrowed). C) Metal-bearing fluid alteration textures and chaotic secondary vein fabrics which may leach elements into their immediate vicinity. (dark and stained regions). D-E) Stylolites rich in metal oxides of probable syn- or post-diagenetic origin and large secondary oxide particle of no relevance to the primary sedimentary fabric. F) Primary laminated chert altered and distended by secondary quart veins. Note the disruption of primary carbonaceous laminations (arrowed). G) Irregular clots in direct contact with stylolites (cut across), considered unreliable for analysis (red arrow). Clots further from the stylolite are considered fit for analysis (green arrow). H) Carbonaceous material entrained within post-depositional megaquartz vein (arrowed), the primary character of which cannot be assured; therefore, these are considered unsuitable for analysis.

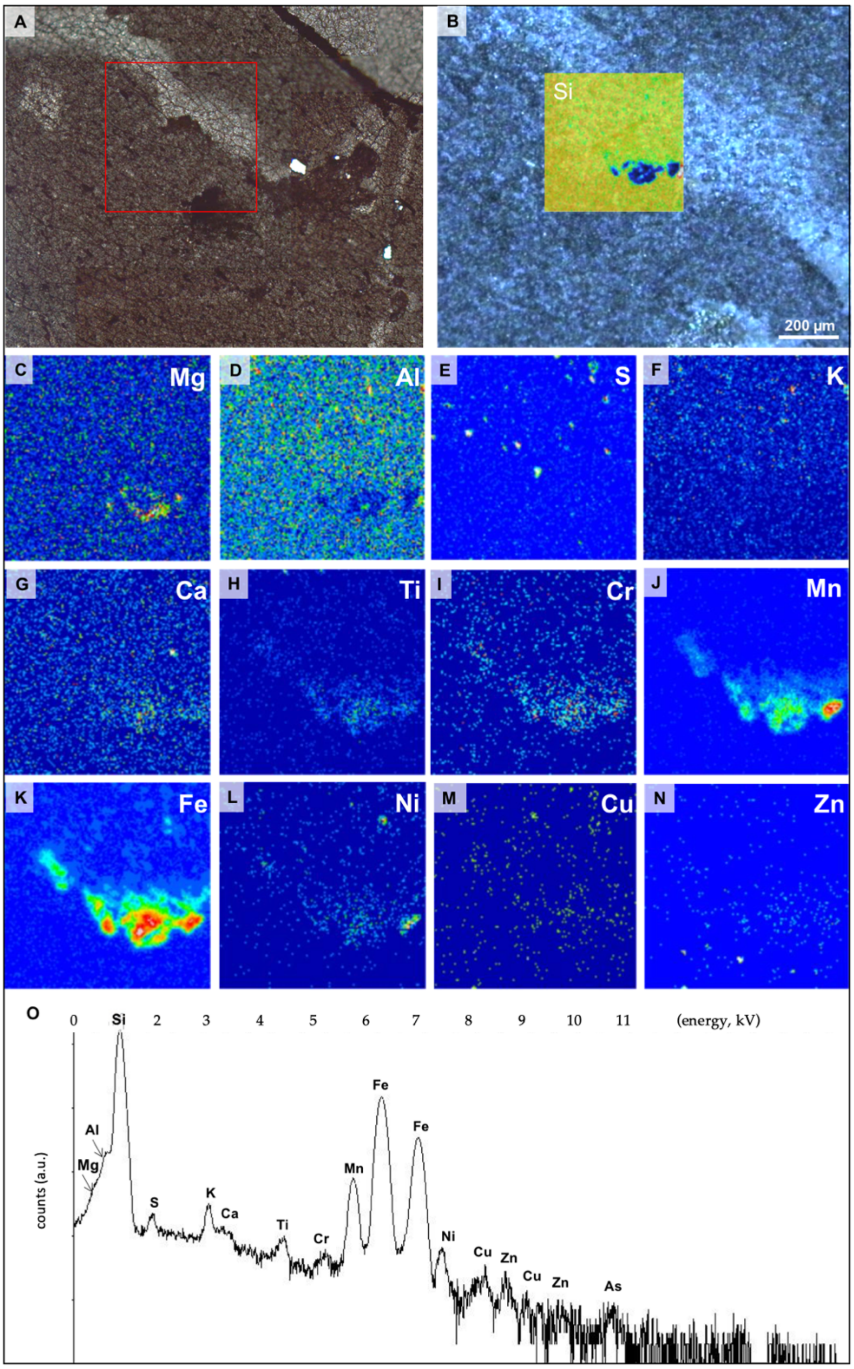

Figure S10. PIXE analysis of one matrix zone (sample 12SA34). **A**) Transmitted light photomicraph mosaic of the matrix. Red box indicates the region of B. **B**) Optical image of the matrix, superimposed with silica map, demonstrating the anti-correlation with the siderite-rhodochrosite crystals. **C**) Elemental maps of major and trace elements within the matrix: Mg, Ca, Ti, Cr, Mn, Fe, Ni, Cu and Zn are concentrated within the siderite-rhodochrosite crystal aggregate, Al is anti-correlated with the crystals. Fe, Ni, Cu and Zn are further concentrated in micron-scale particles elsewhere in the matrix. **D**) Corresponding PIXE spectrum.

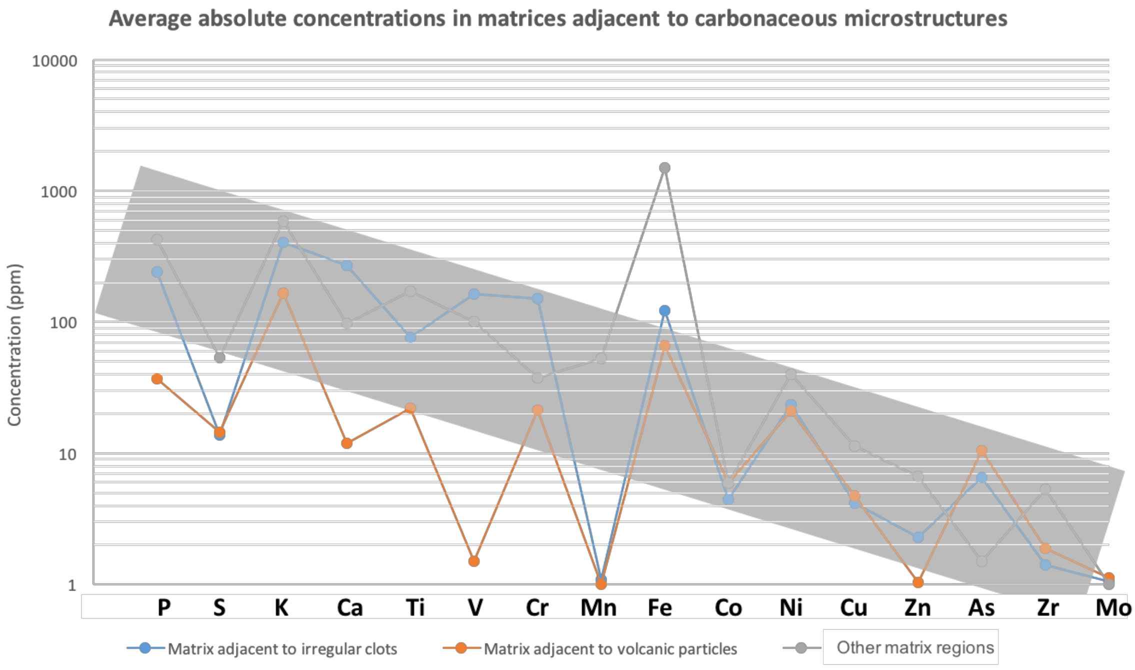

Figure S11. Average concentrations of the sixteen studied elements within the matrices adjacent to the carbonaceous microstructures of interest. Concentrations are broadly equivalent, with the exception of greatly enriched Fe in sample 12SA34 (matrix adjacent to flakes), which can be accounted for by the presence of randomly distributed siderite. The grey trend-line is comparable to that of the ICP-MS data (Fig. 3A) and all trends bear similarity to the flakes trend-line (Fig. 3D), indicating no directional concentration by the flakes.

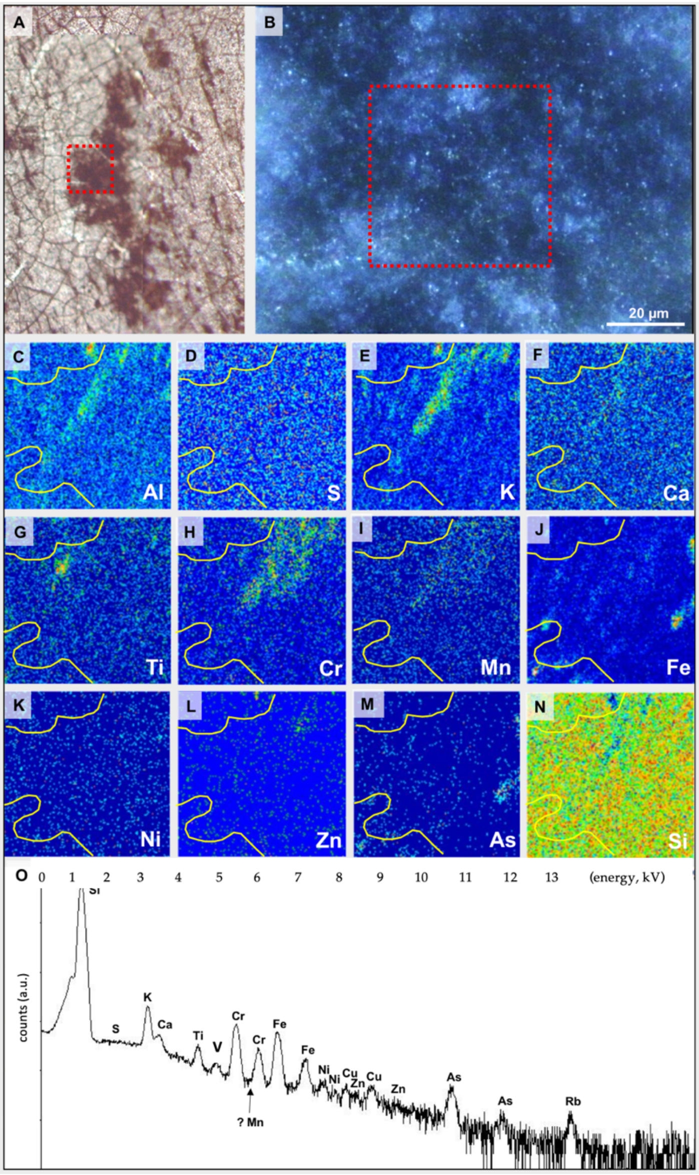

Figure S12. PIXE analysis of irregular clot (sample 99SA07). **A**) Transmission light photomicrograph mosaic of the studied clot. Red box indicates the region of B. **B**) Optical image of the clot. Red box indicates the region mapped. **C**) Elemental maps of major and trace elements within the clotted material and adjacent matrix (yellow lines denote the edge of the carbon). Note that, aside from enrichment in the linear crystal mass at the upper centre of the image (and other minor minerals), Al, S, K, Cr, Mn, Fe, Ni, Zn and As are broadly enriched within the carbonaceous matter relative to the matrix (compare matrix zone at upper left). Ca and Ti are uniformly enriched throughout both clot and matrix. Si is depleted within the clotted carbon. **D**) Spectrum from the zone of carbonaceous material. Although Mn is mapped, its presence is difficult to determine, since it occurs on the shoulder of the Cr K_{beta} peak. X and Y axes are energy channel (instrumental) and counts per channel, respectively.

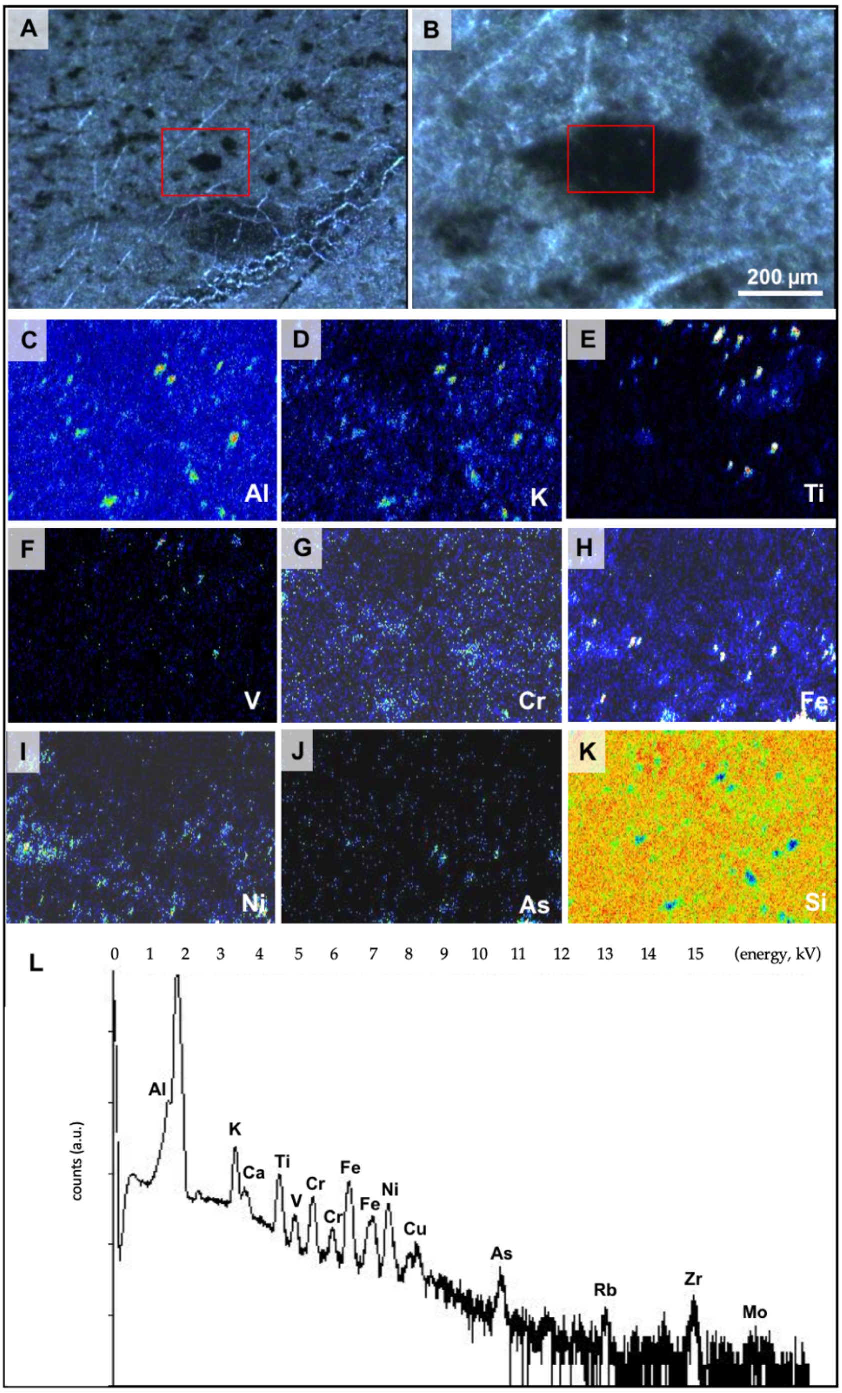

Figure S13. PIXE analysis of carbon-coated volcanic particle (sample 14SA01). **A**) Optical image of the sample. Red box indicates the region of B. **B**) Optical image of the particle. Red box indicates the region mapped. **C**) Elemental maps of major and trace elements within the carbonaceous material. **D**) Spectrum from the zone of carbonaceous material. X and Y axes are energy channel (instrumental) and counts per channel, respectively.

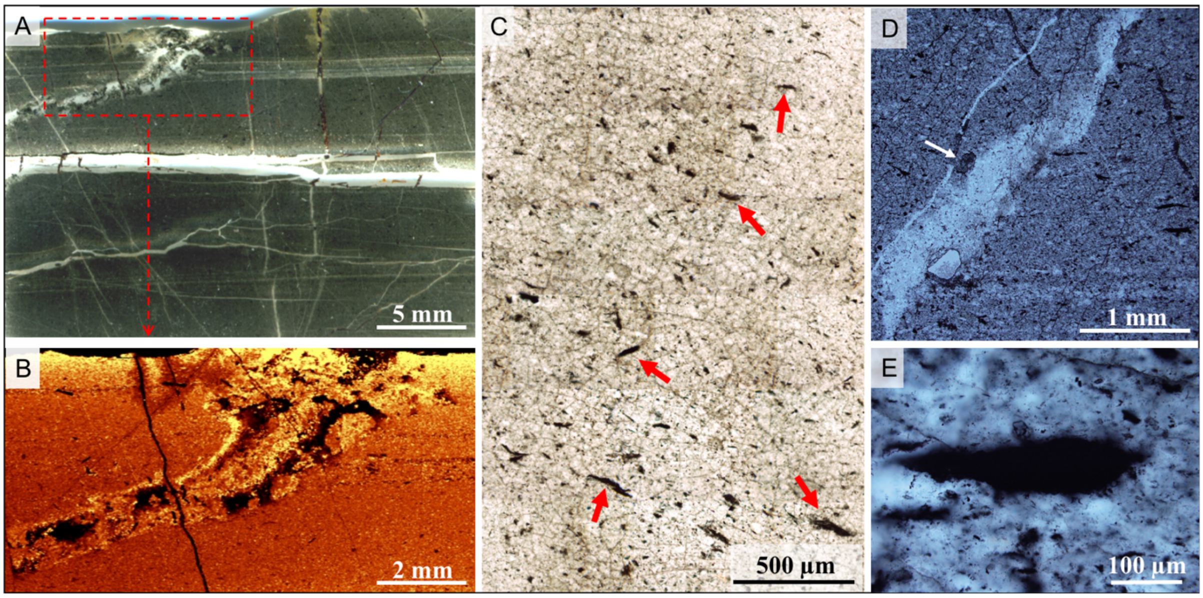

Figure S14. Geological setting of carbonaceous flakes in hydrothermally permeated sediment. **A**) Thin section scan of sample 12SA34, through which pass vertical and stratiform veins of hydrothermal origin. The red box denotes a miniature vent mapped by Raman spectroscopy in B. **B**) Raman map of carbon, wherein brightness corresponds to occurrence of carbonaceous material. Note therefore that carbon is greatly enriched within the fluids in the vent relative to the surrounding sediment. **C**) Carbonaceous flakes (arrowed) and disaggregated carbonaceous material distributed throughout the matrix. **D**) PIXE instrument's optical image of the matrix, which includes frequent siderite particles (arrowed). **E**) PIXE instrument's optical image of a carbonaceous flake with tapered margins.

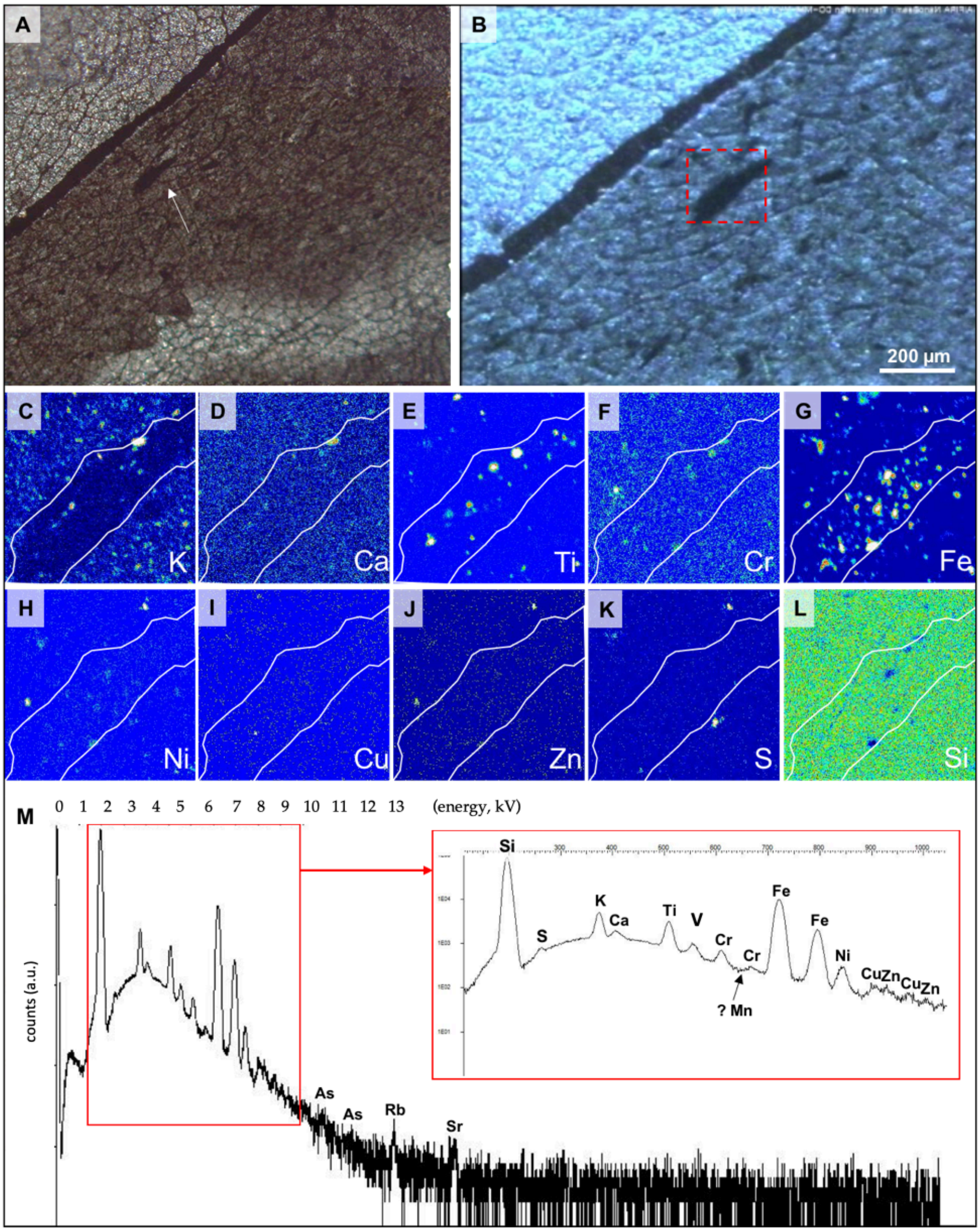

Figure S15. PIXE elemental mapping of flake (sample 12SA34). **A**) Petrographic image mosaic of the studied flake. **B**) PIXE instrument's optical image of the clot. Red box indicates the region mapped. **C**) PIXE spectroscopy mapping of major and trace elements within the flake and adjacent matrix (yellow lines denote the edge of the carbon). K, Ti, Cr, Fe, Ni and Cu are enriched specifically in micron-scale mineral particles within the flake and matrix. Zn and S are enriched specifically in particles dispersed throughout the matrix. Ca is uniformly enriched throughout both flake and matrix. **D**) PIXE spectra from the zone of carbonaceous material. The presence of Mn in the spectra is difficult to determine, since it occurs on the shoulder of the minor Cr K_{beta} peak.

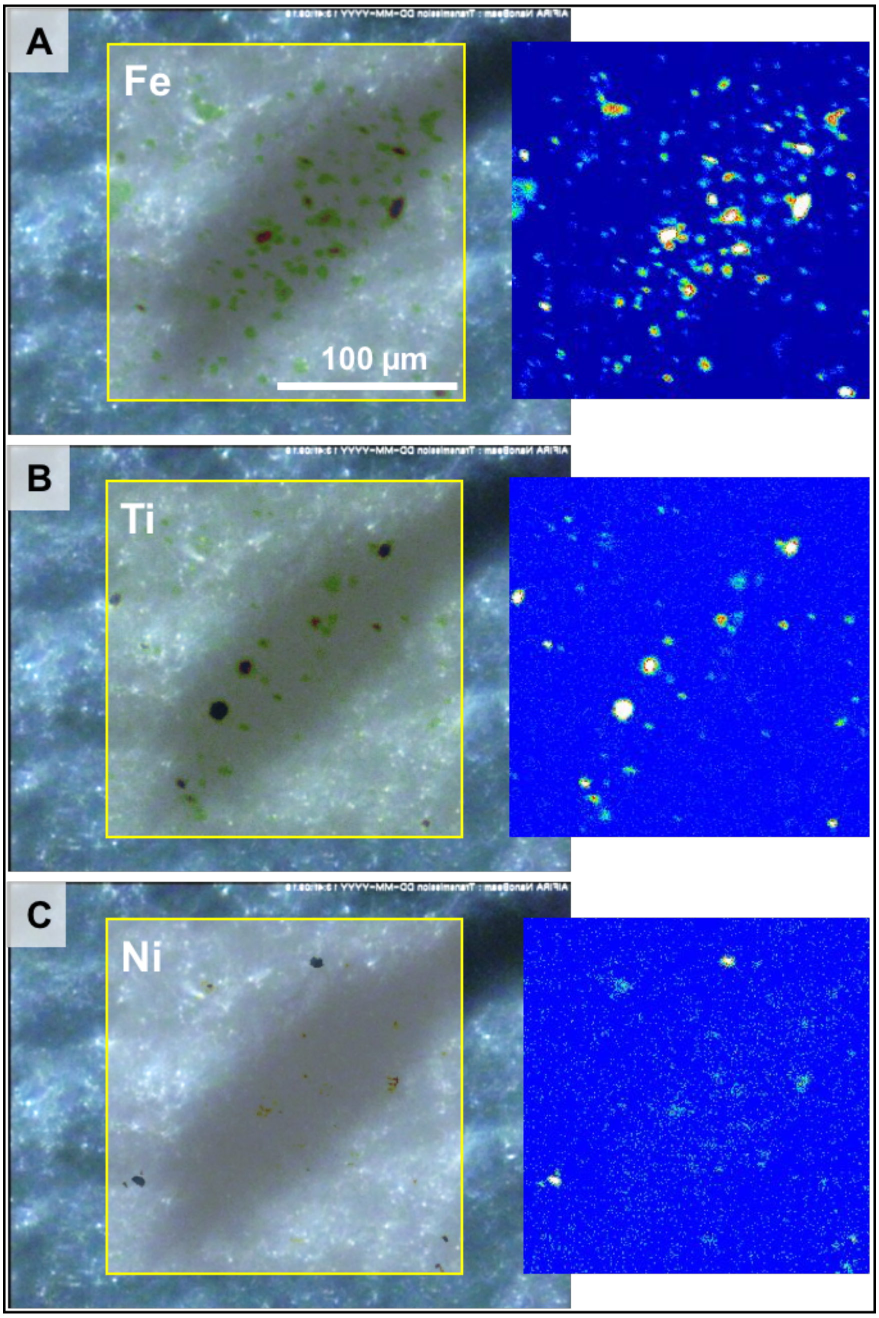

Figure S16. PIXE elemental mapping of **A**) Fe, **B**) Ti and **C**) Ni within carbonaceous flake (sample 12SA34). These maps (comparable in the case of each flake studies) demonstrate beyond doubt that the enrichment within this morphology of carbonaceous material is not due to trace metals being distributed throughout the carbon, but rather localised within equant microcrystals.

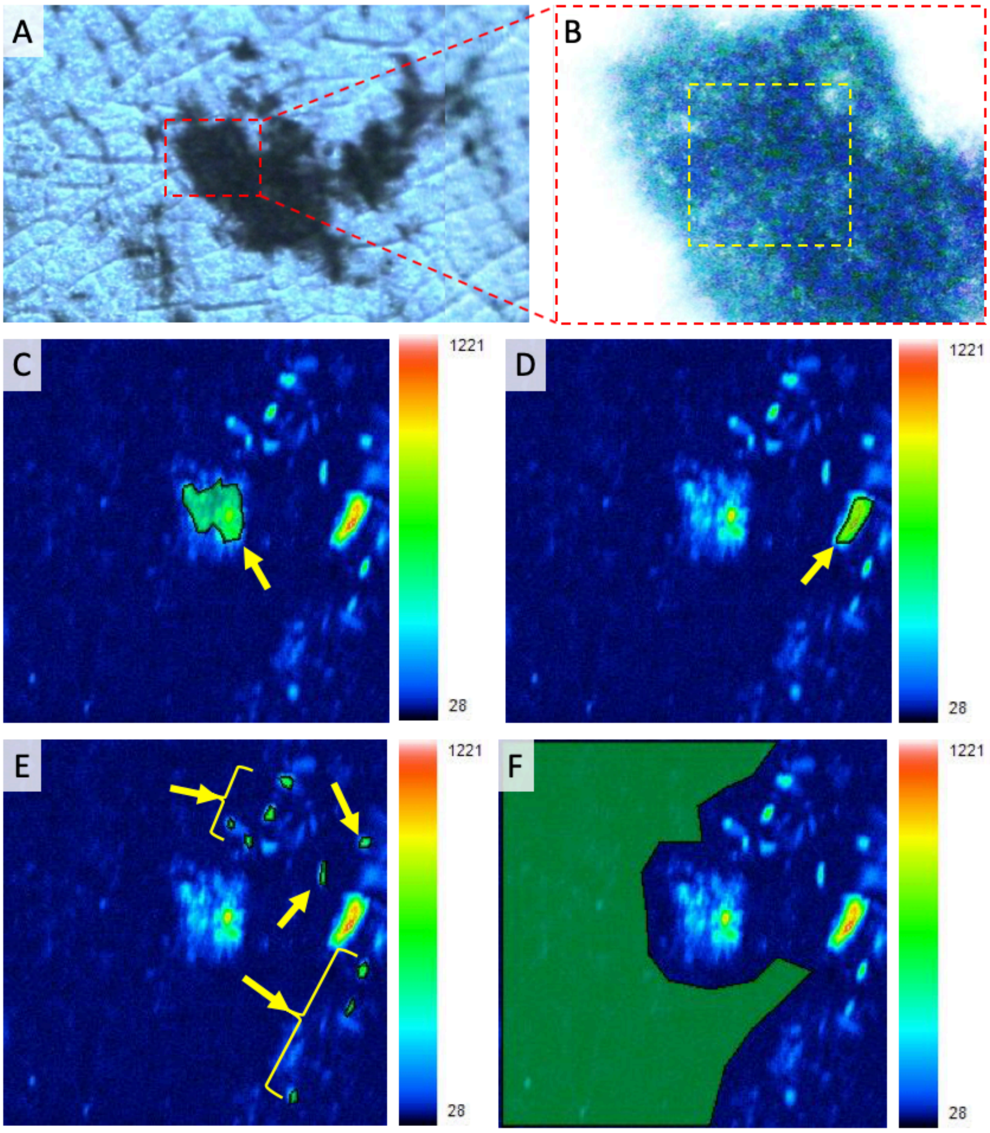

Figure S17. Application of the ‘mineral effect’, identification of micron-scale mineral phases and analysis of the selected area from which the elemental complement of carbonaceous material was calculated using PIXE software. **A-B**) μPIXE instrument optical views of a section of the studied clot. Region of analysis shown by yellow box = 200 μm square). **C-E**) Yellow arrows indicating the presence of mineral particles that were eliminated from analysis of carbonaceous material, since they may skew the results of metal enrichment to higher values. **F**) Example area of carbonaceous material characterised by μPIXE, which is demonstrably free of mineral particles. Consequently, all metal enrichment reported in this region will be indigenous to carbonaceous material. The reported results show only the analyses from carefully selected and strictly characterised areas such as these.

Table S1. Results ($\delta^{13}C$) of SIMS carbon isotope analyses.

Analysis ID	Ratio Average	SE%	$\delta^{13}C$ (PDB) ‰	$\delta^{13}C$ ± ‰
Sample 99SA07				
Irreg. Clot 1	0.01067	0.11	-12.65	2.04
Irreg. Clot 2	0.01064	0.24	-15.86	2.95
Irreg. Clot 3	0.01065	0.11	-15.15	2.04
Irreg. Clot 4	0.01077	0.09	-3.49	1.94
Irreg. Clot 5	0.01071	0.11	-9.21	2.04
Irreg. Clot 6	0.01066	0.15	-14.33	2.28
Irreg. Clot 7	0.01075	0.17	-5.27	2.42
Irreg. Clot 8	0.01079	0.15	-1.47	2.28
Sample 12SA16				
Irreg. Clot 9	0.01066	0.17	-13.44	2.42
Irreg. Clot 10	0.01060	0.29	-19.54	3.37
Irreg. Clot 11	0.01079	0.10	-1.89	1.99
Irreg. Clot 12	0.01070	0.15	-10.54	2.28
Irreg. Clot 13	0.01067	0.07	-13.42	1.85
Sample 12SA09				
Irreg. Clot 14	0.01073	0.15	-6.90	2.28
Irreg. Clot 15	0.01071	0.09	-8.83	1.94
Irreg. Clot 16	0.01066	0.17	-14.25	2.42
Irreg. Clot 17	0.01077	0.14	-3.67	2.21
Irreg. Clot 18	0.01081	0.09	0.03	1.94
Sample 12SA09				
Coating 1	0.01037	0.91	-41.30	9.26
Coating 2	0.01070	0.17	-10.14	2.42
Coating 3	0.01071	0.24	-9.19	2.95
Coating 4	0.01062	0.27	-17.51	3.20
Coating 5	0.01070	0.18	-10.24	2.49
Coating 6	0.01063	0.21	-16.77	2.71
Coating 7	0.01057	0.28	-22.81	3.28
Sample 14SA01				
Coating 8	0.01060	0.25	-19.67	3.03
Coating 9	0.01067	0.17	-12.63	2.42
Coating 10	0.01074	0.31	-5.99	3.54
Coating 11	0.01071	0.12	-9.47	2.09
Coating 12	0.01076	0.16	-4.70	2.35
Coating 13	0.01062	0.20	-18.12	2.64
Coating 14	0.01070	0.19	-9.71	2.56
Coating 15	0.01064	0.17	-15.88	2.42
Coating 16	0.01064	0.29	-15.58	3.37
Coating 17	0.01061	0.17	-18.84	2.42
Coating 18	0.01065	0.24	-14.38	2.95

Sample 99SA07				
Other CM 1	0.01056	0.45	-23.89	4.82
Other CM 2	0.01089	0.13	7.95	2.15
Other CM 3	0.01084	0.11	3.43	2.04
Other CM 4	0.01083	0.15	2.22	2.28
Other CM 5	0.01073	0.2	-6.95	2.64
Other CM 6	0.01069	0.14	-11.49	2.21
Sample 12SA36				
Other CM 7	0.01095	0.11	13.51	2.04
Other CM 8	0.01074	0.29	-5.94	3.37
Other CM 9	0.01076	0.16	-4.48	2.35
Other CM 10	0.01078	0.23	-2.28	2.87
Other CM 11	0.01070	0.44	-9.71	4.72
Other CM 12	0.01033	3.72	-45.01	37.24
Sample 14SA01				
Other CM 13	0.01031	0.81	-47.44	8.28
Other CM 14	0.01057	0.28	-22.81	3.28
Other CM 15	0.01066	0.24	-14.19	2.95
Other CM 16	0.01069	0.14	-11.49	2.21

Table S2. µPIXE quantification: elemental concentrations within carbonaceous materials

Element	Irregular clot 1	Irregular clot 2	Irregular clot 1	Irregular clot 1	Irregular clot 1	Particle coating 1	Particle coating 1	Particle coating 1	Particle coating 1	Flake 1	Flake 2	Flake 3	Flake 4	Flake 5	Flake 6
P	1654	304	207	71	45	0	61	0	0	253	244	75	32	3132	1490
S	603	0	48	4	0	0	223	41	0	24	67	287	17	64	65
K	2030	30	208	81	176	0	430	339	0	364	17	796	56	101	519
Ca	84	2	5	0	9	0	0	0	0	15	79	39	156	160	240
Ti	62195	160	199	48	56	0	21	0	0	1085	7206	534	117	59	355
V	1059	337	228	0	2	39	5	36	245	75	240	158	134	0	112
Cr	1906	169	158	57	0	0	51	35	0	9	213	17	0	0	37
Mn	12	0	2	0	0	0	0	0	0	6	63	0	15	0	645
Fe	5120	243	186	263	185	163	0	67	223	4161	41102	1459	632	1651	3491
Co	3	9	12	80	4	0	35	0	0	0	0	29	0	46	0
Ni	14	44	37	392	32	1	874	257	118	50	451	81	18	23	49
Cu	102	8	2	0	4	0	605	0	26	19	4	7	12	11	0
Zn	23	6	4	0	1	0	5	0	0	3	21	10	0	7	7
As	139	7	19	2	48	0	89	0	0	0	0	8	0	0	0
Zr	108	0	1	0	2	0	0	25	0	29	105	0	0	0	19
Mo	1	0	1	0	0	0	0	0	0	0	0	0	0	0	0

Table S3. µPIXE quantification: elemental concentrations within the adjacent matrix Regions of analysis were chosen adjacent to the microstructures analysed in Table S2.

Element	Irregular clot 1	Irregular clot 2	Irregular clot 1	Irregular clot 1	Irregular clot 1	Particle coating 1	Particle coating 1	Particle coating 1	Particle coating 1	Flake 1	Flake 2	Flake 3	Flake 4	Flake 5	Flake 6
P	0	246	185	1137	22	22	67	22	22	179	406	0	577	1377	0
S	0	0	0	50	0	0	41	0	0	13	34	0	168	40	68
K	712	22	195	1423	99	99	299	99	99	868	129	827	211	1043	461
Ca	73	1582	23	166	15	15	6	15	15	28	41	47	7	74	385
Ti	80	21	90	278	4	4	58	4	4	137	171	221	31	342	128
V	95	64	192	786	1	1	2	1	1	8	28	33	25	309	207
Cr	213	55	240	488	3	3	58	3	3	82	10	24	8	80	23
Mn	0	0	2	0	0	0	0	0	0	2	3	310	0	0	0
Fe	250	99	71	229	42	42	116	42	42	1166	2099	2294	401	1469	1596
Co	0	0	0	10	0	0	16	0	0	5	5	0	0	24	0
Ni	7	1	17	77	5	5	54	5	5	27	36	36	18	21	102
Cu	9	0	4	0	0	0	12	0	0	10	13	5	2	8	30
Zn	8	1	1	3	0	0	1	0	0	3	8	7	3	14	5
As	9	3	0	0	12	12	7	12	12	2	1	2	1	0	0
Zr	1	0	0	0	0	0	4	0	0	2	20	6	0	0	0
Mo	0	0	0	0	0	0	1	0	0	0	0	0	0	0	0

Supplementary Discussion

Validating the interpretation of the elemental signatures observed in carbonaceous material as palaeo-metallomic signatures

1. Palaeoenvironmental calibration

The mode of occurrence of metal enrichment in carbonaceous material is critically important in distinguishing its origin. The palaeoenvironment must be demonstrated to be habitable and, in the event of interpreting metal enrichment as a palaeo-metallomic signature, the predicted organisms and metabolisms should be consistent with the estimated environment.

Highly detailed field observations coupled with bulk and *in situ* geochemical data for the Josefsdal Chert indicate that the environment of deposition was a volcano-hydrothermal basinal setting (Westall et al., 2001, 2006, 2015; Hofmann et al., 2013; Hubert, 2015; this study). Sedimentary structures indicate the action of waves and currents, particularly in banded cherts (Fig. 2A-B), whereas relict mineralogy clearly indicates volcanic activity (Figs. 2E-F; S4-S8), which may have occurred in pulses (Westall et al., 2015). Highly siliceous rocks with poorly laminated macrostructure are best interpreted as chemical precipitates (Fig. 2C-D). Brecciation and distending of bedding planes has been interpreted as the result of the hydrothermal activity that would have accompanied volcanism (Hofmann et al., 2013; Westall et al., 2015). Rare earth element plus yttrium (REE+Y) plots normalised to Mud from Queensland (MuQ) are the appropriate method by which to analyse the relative contributions of chemically distinct fluid reservoirs to Archaean sediments (Kamber et al., 2005; Gourcerol et al., 2015). In these Josefsdal Chert sediments, positive La and Y anomalies (*Eqs. 1, 3*) in bulk ICP-MS analyses testify to the presence of marine inputs, as does the enrichment of heavy REE over light REE (Fig. 2A). Weakly to strongly positive Eu anomalies (up to 2.02; *Eq. 2*) can only be attributed to hydrothermal inputs. Although some Eu enrichment can be attributed to volcanic particle (i.e., feldspar) sequestration of REE (Kerrich et al., 2013), anomalies higher than 1.20 cannot be accounted for through this alternative explanation. Since even small quantities of clastic- or mineral-related input can affect the REE+Y signal of hydrogenous chert, we followed the rigorous approaches of Bolhar and Van Kranendonk (2007), Sugahara et al. (2010) and Bolhar et al. (2015) in testing whether clastic or terrigenous components can be considered an input, and not contamination, to the palaeoenvironment. For each formation, we tested multiple parameters (La, Eu, Ce and Y anomalies and Y/Ho ratios) against total REE+Y, Ti content, and against one another, as a proxy for contamination of the signal by clastic input or other hydrospheric influences. We found no systematic relationships between any two such parameters, ergo 'contamination' by clastic material does not affect the REE+Y signals. Our REE+Y compositions are thus primary. Since similar values are obtained for various parameters (e.g. anomalies) irrespective of the Ti content, fluid chemistry rather than clastic input is the dominant control on REE+Y composition (Bolhar et al., 2015).

Eq.1 $La/La^*_{MUQ} = La_{MUQ}/(Pr^*_{MUQ}(Pr_{MUQ}/Nd_{MUQ})^2)$

Eq. 2 $Eu/Eu^*_{MUQ} = Eu_{MUQ}/(Sm^2_{MUQ} * Tb_{MUQ})^{1/3}$

Eq. 3 $Y/Y^*_{MUQ} = Y_{MUQ}/(0.5Er_{MUQ} * 0.5Ho_{MUQ})$

The major environmental parameters are therefore hydrogenous, of marine and hydrothermal origins. The shallow gradient is indicative of elevated light REE input, which is most plausibly interpreted as the result of terrigenous contributions, i.e., the depositional environment was semi-restricted. Varying anomaly values indicate the fluctuating importance of these reservoirs during the deposition of the Josefsdal Chert. The presence of hydrothermal

fluids in the depositional palaeoenvironment indicates that it would have been a thermophilic setting. High concentrations of LREE and trace elements more generally denote the elevated influence of volcanic and terrigenous inputs. In the Palaeoarchaean, most exposed landmasses were probably of volcanogenic origin (Lowe and Byerly, 1999; Kamber, 2015; Cawood et al., 2018). High concentrations of Fe- and Mn-oxyhydroxides in semi-restricted basins were able to concentrate REE+Y elements and make these elements bio-available (see Jeandel and Oelkers, 2015). The environment of the Josefsdal Chert was therefore metal-rich, thermophilic, and replete with chemical disequilibria as a result of the confluence of fluids of different origins. The studied samples thus reflect a habitable realm.

Further information regarding the stratigraphy and sedimentology of the Josefsdal Chert is given in Westall et al. (2015), including full descriptions of the different lithofacies present and their interrelationships throughout the stratigraphy.

2. Multi-scalar modes of occurrence of carbonaceous material

In light of previous controversies concerning the origin of putatively biogenic carbonaceous material in Archaean meta-sediments (Brasier et al., 2002, 2006; Lindsay et al., 2005; Wacey et al., 2016, 2018), it is imperative to explain the origin of carbonaceous material in terms of its mode of occurrence within the parameters of the environment. For example, Brasier et al. (2002, 2006) and Lindsay et al. (2005) suggested that the Fischer-Tropsch-type reactions, known to occur in natural hydrothermal settings, are potential sources of the carbonaceous material constituting putative biosignatures. Given the evident presence of hydrothermal inputs to the environment of deposition of the Josefsdal Chert, the contribution of abiogenic organic matter to the eventual carbonaceous material reservoir must be appraised. Additionally, Wacey et al. (2018) identified tephra with strong morphological resemblance to putatively fossiliferous material. Due to the strong volcanic inputs to the Josefsdal Chert (Hofmann et al., 2013; Westall et al., 2015), this possibility of carbon accumulation by abiotic means must also be evaluated.

The lack of continuity of the metal-rich signature is a function of the preservation of carbonaceous material. The distribution of elements within PIXE maps is also inconsistent throughout CM (i.e., over micrometric scales; Fig. S17), but this is an authentic representation of the micrometric distribution of the CM, which is dispersed as interleaved fragments between silica crystals after Ostwald ripening under lower greenschist facies metamorphic pressures and temperatures (van Zuilen et al., 2012; Hickman-Lewis et al., 2017). This distribution is further evident from the spatial distribution of point enrichments CM obtained by SIMS.

Transmission electron microscopy observations confirm that this distribution of carbon is responsible for the diffuse appearance of clotted carbon when observed at high magnification in thin section. The 'blackness' of clots/coatings is an optical effect, as demonstrated by high-magnification TEM micrographs (Fig. 2G-H). Where CM is present as only a thin layer, it is evident that the clot is composed of CM interleaved between micro-crystals of quartz, forming a web-like 3D network. The CM itself is near-pure carbon and does not incorporate nanoscopic mineral particles of any mineral other than quartz. Since the quartz lattice is not able to incorporate either trace and rare earth element or transition metals, any indigenous metal-rich signature is only due to that conserved within CM.

Raman spectroscopy indicates that the CM studied herein has undergone some graphitisation (Fig. S3) but is mostly disordered i.e., the D band is predominant over the G band. The thermal maturity of the carbonaceous material is identical to that of the host rock, proving the syngenicity of the former with the latter (after Hubert, 2015; Westall et al., 2015; Hickman-Lewis et al., 2016).

3. Biotic or abiotic origin for carbonaceous material?

In this approach to palaeo-metallomics in deep time, a highly conservative methodology is required. This necessarily involves discounting all potential abiotic means of accumulating both the carbonaceous material and its metal enrichments. Indeed, there are several potential abiotic means of accumulating CM in volcano-hydrothermal environments as seen in the studied samples that deserve consideration:

- Fischer-Tropsch-type organic matter synthesised within the hydrothermal system, extruded together with hydrothermal fluid and sequestered within a gel-like chemical sediment (Lindsay et al., 2005) or adhered to mineral phases (cf. Wacey et al., 2016);
- Detrital organic matter of unknown origin aggregated in the water column, falling out of suspension and accumulating within a gel-like sediment;
- Detrital organic matter of mixed origins that re-enters into the hydrothermal system, is assimilated within the hydrothermal fluid and redeposited into a chemical sediment with a direct genetic link to the hydrothermal system (the hydrothermal pump hypothesis; Duda et al., 2018);
- Pyrobitumen inclusions generated either during hydrothermal alteration or subsequent metamorphic processes and fluid migration events, which may be Archaean or younger (cf. Buick et al., 1998);
- Carbon-rich volcanic clasts or tephra, naturally deposited in the system, that strongly resemble microfossils or rip-up clasts from microbialites (Wacey et al., 2018);
- Meteoritic carbon exogenously delivered to the shallow-water depositional environment, before being sequestered into a gel-like sediment.

Addressing these possibilities in turn:

Fischer-Tropsch-type products relevant to this abiogenicity debate are hydrocarbons including alkanes, alkenes and alcohols. Such carbonaceous material may aggregate in the vicinity of hydrothermal vents since these reactions are observed to occur in natural hydrothermal settings (McCollum et al., 1999; Sherwood Lollar et al., 2002). The carbon isotopic fractionation in naturally produced liquid hydrocarbons ranges between +40‰ and –80‰, depending upon the carbon source and reaction series followed (McCollum and Seewald, 2006; Taran et al., 2007). The majority of FTT products exhibit strongly negative carbon isotopic fractionations between –40 and –70‰. The probability maxima of the datasets in the aforementioned publications form a continuum of maxima throughout the values from +40 to –80‰, with striking probability maxima at highly depleted values, corresponding to the abiogenic production of methane by one suite of FTT reactions (McCollum and Seewald, 2006). The FTT reactions would not yield the relatively restricted range of values mostly between –10 and –21‰ reported herein (Fig. 3; Tables 1-2). It is also unknown for FTT processes to produce CM with recurrent morphologies such as the clots and coatings described in this manuscript. Abiotic CM would instead be expected to form unremarkable and nondescript aggregations of material, since it has no morphogenetic growth framework. It is highly unlikely that CM originating from the FTT reactions would form either particle-specific, non-isopachous carbonaceous coatings or recognisable irregular, stellate clots in multiple sedimentary layers. For these reasons, both isotopic and morphogenetic, we consider it entirely unlikely that the CM herein is the result of FTT hydrocarbon production. While it is likely that the FTT processes were active in this environment, their signal is not dominant, likely because their products have very minor prevalence in comparison to other CM.

Detrital organic matter falling out of suspension after autotrophic-heterotrophic production and consumption in the water column may be the source the particulate organic material characterising some CM-rich layers in Archaean cherts (Walsh, 1992; Walsh and

Lowe, 1999). Sub-rounded, fluffy particles of CM – superficially similar to the clots described herein – have been identified in the stratiform 'Apex chert' (Brasier et al., 2011; Hickman-Lewis et al., 2016) and the Dresser Formation (Wacey et al., 2018; Duda et al., 2018; Igisu et al., 2018). For the latter case, a contribution from water column productivity has been implied. In modern seafloor sediments, 'marine snow' – to which such influx is analogous – occurs as blanket-like deposits over large areas of the seafloor. This is not the case of the irregular clotted morphologies in the Josefsdal Chert since they occur within spatially restricted facies confined to regions of higher hydrothermal flux (Westall et al., 2015). Nor could this possibly be the case for the particle coating morphologies, which are, as defined by their appellation, particle-specific within individual sedimentary layers. Increased CM-rich layers in the vicinity of the outflow of hydrothermal material is instead consistent with an origin as biogenic colonisation associated with hydrothermal effluent (flocs, cf. Juniper et al., 1995; Crowell et al., 2008). For these organo-sedimentological reasons, it is impossible to interpret the clotted or coating morphologies as detrital organic matter sourced from outside the local environment.

Detrital organic matter reworked and sequestered within hydrothermal veins (Duda et al., 2018) is a further possible interpretation of the origin of this carbon. We do not consider that the clotted or coating morphologies could be reworked material since they occur within primary rock fabrics and far from obvious field- and thin section-scale remobilisation of material. Their morphologies are not consistent with having undergone transport or mechanical erosion in the "hydrothermal pump" described by Duda et al. (2018) or within the framework of the clot-like objects described by Morag et al. (2016). We do not therefore consider it possible to explain the petrographic occurrence of these two clotted textures in terms of detrital CM inputs.

Oil droplets in the form of thermally mature bitumen globules have been reported from Archaean successions (Buick et al., 1998; Rasmussen and Buick, 2000), and such structures could co-exist with clotted carbonaceous material. Extensive and highly detailed SEM and TEM imaging of the CM studied herein shows that they lack both the regular, smooth shapes at the microscale (or the granular mesoscale morphologies) of reported bitumen aggregates and the enrichment of radioactive minerals or elements in their cores. Consequently, the organic material constituting these clots is unequivocally not thermally matured bitumen.

Wacey et al. (2018) provide a compelling account of carbonaceous, entirely abiogenic, volcanic pseudofossils from the Dresser Formation. Observations of carbonaceous laminae and accumulations within and onto shards of altered volcanic glass is a potential origin for the microstructures herein given the notable volcanogenic inputs evidenced by SEM-EDS and Raman spectroscopy observations in Figures S3-S8). It is not possible to compare our findings with the pseudocellular morphologies reported by Wacey et al. (2018) since no such morphologies exist in our samples. The irregular clots bear no morphological similarity to any of the structures in Wacey et al. (2018) and should be considered of different origin. It is more challenging to overcome the possibility that the particle coatings may originate from accumulation on altered volcanic glass and other minerals and we cannot unambiguously discount this possibility. Nonetheless, since the colonisation of volcanic and other particle surfaces in contemporaneous cherts has been noted (Westall et al., 2011; Wacey et al., 2011), it appears that both biotic and abiotic possibilities exist for this fabric, and these should be explored in more detail in individual cases, ideally using a multi-technique assessment as conducted herein.

Meteoritic carbon is the final potential alternative origin for the accumulation of CM seen in the Josefsdal Chert. The volume of CM exogenously delivered to the early Earth was vastly greater than at present due to increased impactor flux early in the history of the solar system (Maurette and Brack, 2006; Koeberl, 2006). Impactor events, in this case carbonaceous chondrites, would result in blanket-like carbon deposition and consistent geochemical signatures on local scales. Examples include the Ir and Pt-group element enrichments seen together with microkrystites in the Marble Bar chert, Pilbara (Glikson et al., 2016), and the

impact spherule layers observed throughout the Barberton greenstone belt (Lowe and Byerly, 2003). Extraterrestrial organic material can exist in Archaean rocks where silicification is sufficiently rapid to preserve diagnostic indications of meteoritic origins (Gourier et al., 2019). No regionally traceable blanket-like aggregations of CM or other geochemical and petrological signatures have been noted in this Josefsdal Chert material, which is in stark contrast to the regionally detectable extraterrestrial geochemical signatures found in higher horizons of the same suite of chert (Gourier et al., 2019). The irregular clot and particle coating microstructures cannot be explained in terms of any known morphology relating to meteoritic impact. While there was likely a consistent meteoritic input of carbon to the Josefsdal Chert and other Archaean environments, its contribution to these particular microstructures was negligible, and likely zero.

The careful and comprehensive consideration above shows that no plausible abiological interpretation for either the clot or coating CM morphologies described herein can be sustained under scrutiny. In all but one case – the possibility that particle coatings may reflect non-biological accumulation of carbon on volcanogenic particles – an abiological interpretation is impossible within any known framework. Since the CM described is present as recognisable microstructures encapsulated rapidly within silica representing the primary deposition of these meta-sediments, and since demonstrable biogenic interpretations exist for carbonaceous accumulations on volcanic particles (Westall et al., 2006, 2011; Furnes et al., 2007), an alternative explanation is required to assess the origin of all CM described herein. The potential biogenic interpretative frameworks are as follows:

- Detrital biogenic material that has been sequestered into a gel-like sediment with a direct genetic link to the adjacent hydrothermal system, and is thus not necessarily a primary fabric;
- Mixed community cellular material and extracellular products that developed *in situ* and were consequently preserved in a manner reflecting their mode of growth (a primary imprint) by virtue of the rapidity of their silicification.

Addressing these possibilities in turn:

As noted above, there is no evidence for the detrital origin of the CM described in this study. Both the fact that the CM morphologies are facies-specific and, within that, particle-specific, features is unequivocally diagnostic of their origin as a primary textural element. Furthermore, the lateral variation of clotted structures denotes that they occur under local environmental control. Previous coupled fieldwork and petrographic work in the Josefsdal Chert has shown that clot-rich samples originate from more highly silicified regions of outcrops that are thought to refelct proximity to hydrothermal veins. Detrital CM, whether of biological or abiological origin, would not form facies-specific microstructures with irregular morphologies, but rather a laterally indiscriminate enrichment throughout all time-equivalent sediments of the Josefsdal Chert basin. We therefore discount one of the possible biological interpretations – that the irregular clots and particle coatings reflect the sequestration of biogenic material from mixed and unknowable proximal and distal sources – as unsupported by our detailed field and petrographic assessment of these rocks.

The second biological interpretation, that the CM represents the rapidly preserved primary imprint of a community that developed *in situ* within certain horizons of the Josefsdal Chert, requires several key pieces of evidence. Firstly, it demands that the structures, being rapidly silicified, should be preserved in three dimensions with no preferential axis. The carbonaceous matter forming both irregular clots and particle coatings is indeed preserved in three dimensions, as demonstrated by thin sections cut both parallel and perpendicular to the faint bedding present in these samples. Furthermore, the clots are apparently templated by

micron-scale minerals which may or may not occur within the carbonaceous matter, and do not contribute to grain-supported fabrics in the cherts (as would be the case for pelagic marine snow or detrital carbonaceous material deposits). Secondly, the clots and coatings should be facies-specific, suggesting an ecological functionality, and not be ubiquitous throughout the Josefsdal Chert sediments, which are highly varied. Both microstructures are indeed lithofacies-specific, occurring only in near-pure hydrothermally influenced chert for irregular clots, and within hydrothermally influenced, shoreface volcaniclastic sediments for particle coatings. The first of these is not a common lithofacies within the Josefsdal Chert (see stratigraphic column from Westall et al., 2015), and this is compelling evidence for the irregular clots being an environmentally constrained phenomenon. As noted above, this lithofacies is also laterally variable. The origin of biological morphological specificity under facies influence has been noted in mat-building fabrics in younger sediments from the Mesoarchaean Moodies Group of South Africa (Homann et al., 2015). Concomitantly, one would certainly not expect palaeoenvironmental or facies restriction for detrital carbonaceous matter of either biogenic or abiotic origin. This further argues for the rejection of those hypotheses.

If the clotted carbon reflects a community signature as seen in modern-day microbe-mineral clots and flocs (Juniper et al., 1995; Crowell et al., 2008), and not a monospecific entity (which is highly unlikely and impractical in most natural systems), the carbon isotopic fractionation should be varied and centred around values that are negative. Most common anoxygenic photosynthetic bacteria produce carbon isotope fractionations between –8 and –22‰ (Schidlowski et al., 1984, 1988). This almost exactly matches the isotopic fractionation seen in our samples ($\delta^{13}C$ between –3.5 and –41.3‰, mostly –11.5‰ to –21.0‰, with strong probability maxima between –10 and –21‰). These values are consistent with the expected biomes present on the anoxic early Earth, which were likely mediated in part by RuBisCO (Nisbet, 2000; Nisbet and Sleep, 2001; Arndt and Nisbet, 2012). Values that significantly deviate from these probability maxima are generally not of significant number; the overwhelming majority of $\delta^{13}C$ measurements (even in non-specific morphologies of carbon) are between 0 and –25‰, which is highly consistent with anoxygenic, sulphate-reducing and methane-metabolising organisms.

We are therefore able to both refute all potential abiological (and some unsupported biological) hypotheses on the origin of these microstructures and arrive at a conclusion for their origin that satisfies two- and three-dimensional petrographic observations and geochemical data from isotope ratio mass spectrometry. This conclusion is consistent with expected biomes in the palaeoenvironment determined for these cherts based on stratigraphy, trace and rare earth element geochemistry and Archaean ecosystem theory (e.g., Nisbet, 2000).

Consequently, it is acceptable to assess the metal enrichments observed by particle-induced X-ray emission (PIXE) in a biological framework of understanding. Indeed, this carbonaceous material can be deemed of likely biological origin from these analyses alone.

4. Determining the origin of metal enrichment

Having demonstrated the highly probable biogenicity of the CM described herein, one can then address the potential contributions of various metabolisms through decoding the palaeo-metallome. This approach is described in the main manuscript, however, some important practical details are provided in this supplement. Metals are bio-essential to all life in various concentrations and fractional contributions, and have widespread utility in metalloproteins, enzymatic co-factors, biosynthetic polysaccharides and heteropolymers, and other molecules (Williams, 1981, 2001; Fraústo da Silva and Williams, 2001; Williams and Fraústo da Silva, 2003; Hosseinzadeh, and Lu, 2016; Moore et al., 2017). This metal and metalloid composition is termed the metallome and can be considered the third element of cell expression alongside the genome (the complete set of genes or genetic material present in a

cell or organism) and proteome (the entire complement of proteins that is or can be expressed by a cell, tissue, or organism). Of the three, the metallome is the only likely candidate for preservation throughout deep time, since biomarkers indicative of the proteome have been shown not to preserve in ancient sediments as previously reported (French et al., 2015), but are instead more readily subjected to extremely rapid degradation and denaturing. In our highly conservative approach to the deduction of the palaeo-metallome, we have followed the recommendations of Phanerozoic fossil metallome studies (Wogelius et al., 2011; Edwards et al., 2014). Since the presence of elevated concentrations of certain elements can be linked to their presence in specific cellular nanomachines in precursor biological materials (Zerkle et al., 2005; Lobinski et al., 2010; Cameron et al., 2012), finding spatially delimited significant enrichments is crucial. For example, previous all-elements analysis (terminology of Haraguchi, 2004) has found spatially restricted enrichments in elements that have been interpreted as bio-functional to the plant cells of fossil leaves (Edwards et al., 2014). The detected composition must be explicable in terms of the metabolism of the proposed organism, its palaeoenvironment (which defines the elemental budget of the ecosystem of which it is a part) and its diagenetic history, although we strongly recommend that samples having been subjected to visible diagenetic effects, for example the highly recrystallised stromatolites of the Dresser Formation (Hickman-Lewis et al., 2019) or the tourmaline-altered stromatolites of the Mendon Formation (Byerly et al., 1986) should be discounted as candidates for palaeo-metallomic studies.

The occurrence of CM as fragments interleaved between microquartz crystals (described in Section 1.2) is sustained by the patterns of observation of elemental concentration seen in μPIXE maps (and indeed for carbon detection in SIMS measurements). Individual regions that are strongly enriched in CM are also those that show high metal enrichment in μPIXE maps. The surrounding silica matrix does not show elevated concentrations of metals. There is no direct correlation between the appearance of concentrations in the maps, which are a function of the number of counts at any specific point, and the concentration (ppm) of that element, which is a function of the detector-specific quantification of that element according to the algorithm of Gupix (see Halden et al., 1995; Campbell et al., 1995; Maxwell et al., 1995). Generally, higher concentrations of elements are seen in regions of visually elevated concentration of CM, although we caution that the 'blackness' of a region of chert should be considered an optical effect, as described using sub-micron TEM micrographs above. Although the detection of some elements is below the theoretical detection limit of PIXE (~100 ppm for most elements), the ability to map their occurrence in discrete energy channels is consistent with their occurrence as shown in the individual element maps, but at relatively low concentrations (< 100 ppm) (Halden et al., 1995; Campbell et al., 1995). These elements should, nonetheless, be considered present.

As shown in SEM-EDS analyses and TEM micrographs, the studied clots contained micron-scale mineral phases but did not contain nanoscopic minerals. Nanoscopic minerals therefore cannot be the 'unseen' source of metal enrichment since they are not present. To remove micron-scale enrichments due to minerals, we excluded any small regions within PIXE scans that showed exceptional enrichments in metals (the 'mineral effect', see Methods). The characterisation of carbonaceous materials was then made only on regions that did not include mineral particles. Individual minerals or groups of minerals were identified in PIXE software and avoided during quantification using SupaVisio and Gupix, such that the analysis of CM itself used only regions free from obvious metal enrichments due to micron-scale mineral phases. The size and frequency of occurrence of micron-scale mineral phases directly corresponds to the frequency with which mineral phases are observed in SEM images. Furthermore, the remaining CM shows a weaker enrichment in metals relative to the matrix, demonstrating the success and applicability of our practical approach to removing irrelevant mineralogical metal enrichments.

Consequently, we have deduced the following: i) the metal signature reported in the study is neither due to nanoscopic mineral phases nor to micron-scale mineral phases, because these are, respectively, not present and mitigated by the 'mineral effect'; and ii) the reported metal enrichments are demonstrably indigenous to the carbonaceous matter. Since we have demonstrated above that the CM is highly likely to be of biological origin and was rapidly preserved, we should interpret the concentrations of metals in CM as a palaeo-metallomic biosignature that reflects the characteristics of the precursor biomass. Two key points are then important in the assessment of the signature as a palaeo-metallomic biosignature:

i) Biological carbonaceous matter readily scavenges metals from the environment during its life processes (this is a fundamental process in all metabolism and cellular catalysis; Fraústo da Silva and Williams, 2001), and has been experimentally demonstrated to considerably outcompete non-biological material in the retention of heavy metals (Schultz-Lam et al., 1993, 1996; Loaec et al., 1998). Bacterial surfaces (both Gram negative and Gram positive) provide loci for the sorption of metals due to the overall anionic charges of the electronegative macromolecules that constitute their cell walls. The use of bacteria as a means to counteract the severe pollutant effects of acid mine drainage is testament to their efficiency in metal sorption. The potential for non-specific binding by extracellular polymers requires an appraisal of the potential presence and relevance of each element in turn. For example, based on evolutionary biology and current understanding of the evolution of metal usage in metabolic networks through time, we suggested in the main manuscript that the enrichment of Cu was not significant of the metallome of the precursor biomass, since Cu became important in the prokaryote metallome only after the GOE.

ii) After deposition and rapid silicification, any post-diagenetic leaching, if indeed it occurs, acts following the death of the organism, i.e., the two processes do not act at the same time (Orange et al., 2009, 2011). Since biological molecules are rich in carboxylate groups, peptidoglycan, teichuronic acid and other heteropolymeric polysaccharides, they are likely to retain metals accumulated during life even after death (Schultz-Lam et al., 1993; Orange et al., 2011). This implies that the metallome is a robust biosignature against diagenesis, and argues for the accuracy of our estimated metallomes in decoding the nature of the Palaeoarchaean ecosystem for which they are relics.

For these reasons, in addition to the consideration and comprehensive rejection of abiogenic means of both accumulating the CM and the metal-rich signatures within it described above, we justify the interpretation of the metallic complement of this CM as of biological origin. The relative contributions of each metal are the result of that metal being essential to the original biomass in some specified concentration. Rapid silicification of the CM provides a 'time capsule' of preservation that assures the survival of the palaeo-metallomic biosignature. This correlation of elements with organisms has been demonstrated for Phanerozoic material (Edwards et al., 2014). Negligible alteration of the biosignature can be envisaged after the death of the organism due to the combination of metal retention expected in biological organic matter and the impermeability of chert to secondary alteration and elemental remobilisation.

References

Arndt, N.T., Nisbet, E.G., 2012. Processes on the young Earth and the habitats of early life. Annual Review of Earth and Planetary Sciences 40, 521-549.

Brasier, M.D., Green, O.R., Jephcoat, A.P., Kleppe, A.K., Van Kranendonk, M.J., Lindsay, J.F., Steele, A., Grassineau, N.V., 2002. Questioning the evidence for Earth's oldest fossils. Nature 416, 78–81.

Brasier, M.D., McLoughlin, N., Green, O., Wacey, D., 2006. A fresh look at the fossil evidence for early Archaean cellular life. Philosophical Transactions of the Royal Society of London B: Biological Sciences 361, 887–902.

Brasier, M.D., Wacey, D., McLoughlin, N., 2011. Taphonomy in temporally unique settings: an environmental traverse in search of the earliest life on Earth. In P.A. Allison and D.J. Bottjer (Eds.), Taphonomy: Process and Bias Through Time, 487 Topics in Geobiology 32, Springer.

Buick, R., Rasmussen, B., Krapez, B., 1998. Archean oil: evidence for extensive hydrocarbon generation and migration 2.5–3.5 Ga. AAPG Bulletin 82, 50–69.

Campbell, J.L., Teesdale, W.J., Halden, N.M., 1995. Theory, practice and application of PIXE microanalysis and SPM element mapping. Canadian Mineralogist 33, 279–292.

Cawood, P.A., Hawkesworth, C.J., Pisarevsky, S.A., Dhuime, B., Capitanio, F.A., Nebel, O., 2018. Geological archive of the onset of plate tectonics. Phil. Trans. R. Soc. A 376, 20170405.

Crowell, B.A., Lowell, R.P., Von Damm, K., 2008. A model for the production of sulfur floc and "snowblower" events at mid-ocean ridges. Geochemistry Geophysics Geosystems 9, Q10T02.

Duda, J.-P., Thiel, V., Bauersachs, T., Mißbach, H., Reinhardt, M., Schäfer, N., Van Kranendonk, M.J., Reitner, J., 2018. Ideas and perspectives: hydrothermally driven redistribution and sequestration of early Archean biomass – the "hydrothermal pump hypothesis". Biogeosciences 15, 1535–1548.

Edwards, N.P., Manning, P.L., Bergmann, U., Larson, P.L., van Dongen, B.E., Sellers, W.I., Webb, S.M., Sokaras, D., Alonso-Mori, R., Ignatyev, K., Barden, H.E., van Veelen, A., Anné, J., Egerton, V.M., Wogelius, R.A., 2014. Leaf metallome preserved over 50 million years. Metallomics 6, 774–782.

Fraústo da Silva, J.J.R., Williams, R.J.P., 2001. The biological chemistry of the elements. Oxford University Press, pp. 600.

French, K.L., Hallman, C., Hope, J.M., Schoon, P.L., Zumberge, J.A., Hoshino, Y., Peters, C.A., George, S.C., Love, G.D., Brocks, J.J., Buick, R., Summons, R.E., 2015. Reappraisal of hydrocarbon biomarkers in Archean rocks. PNAS 112, 5915–5920.

Furnes, H., Banerjee, N.R., Staudigel, H., Muehlenbachs, K., McLoughlin, N., de Wit, M., Van Kranendonk, M., 2007. Comparing petrographic signatures of bioalteration in recent to Mesoarchean pillow lavas: tracing subsurface life in oceanic igneous rocks. Precambrian Research 158, 156-176.

Glikson, A.Y., Hickman, A., Evans, N.J., Kirkland, C.L., Park, J.-W., Rapp, R., Romano, S., 2016. A new ∼3.46 Ga asteroid impact ejecta unit at Marble Bar, Pilbara Craton, Western Australia: A petrological, microprobe and laser ablation ICPMS study. Precambrian Research 279, 103–122.

Gourcerol, B., Thurston, P.C., Kontak, J.D., Côté-Mantha, O., 2015. Interpretations and implications of LA ICP-MS analysis of chert for the origin of geochemical signatures in banded iron formations (BIFs) from the Meadowbank Gold Deposit, Western Churchill Deposit, Nunavut. Chemical Geology 410, 89–107.

Halden, N.M., Campbell, J.L., Teesdale, W.J., 1995. PIXE microanalysis in mineralogy and petrology. Canadian Mineralogist 33, 293–302.

Hickman-Lewis, K., Garwood, R.J., Brasier, M.D., Goral, T., Jiang, H., McLoughlin, N., Wacey, D., 2016. Carbonaceous microstructures of the 3.46 Ga stratiform 'Apex chert', Chinaman Creek locality, Pilbara, Western Australia. *Precambrian Research*, 278, 161-178.

Hickman-Lewis, K., Garwood, R.J., Withers, P.J., Wacey, D., 2017. X-ray microtomography as a tool for investigating the petrological context of Precambrian cellular remains. In *Earth System Evolution and Early Life: A Celebration of the Work of Martin Brasier*, Geological Society of London Special Publication 448.

Hofmann, A., Bolhar, R., Orberger, B., Foucher, F., 2013. Cherts of the Barberton Greenstone Belt, South Africa: Petrology and trace-element geochemistry of 3.5 to 3.3 Ga old silicified volcaniclastic sediments. South African Journal of Geology 116, 297-322.

Homann, M., Heubeck, C., Airo, A., Tice, M.M., 2015. Morphological adaptations of 3.22 Ga tufted microbial mats to Archean coastal habitats (Moodies Group, Barberton Greenstone Belt, South Africa). Precambrian Research 266, 47–64.

Hosseinzadeh, P., Lu, Y., 2016. Design and fine-tuning redox potentials of metalloproteins involved in electron transfer in bioenergetics. Biochim. Biophys. Acta Bioenerg. 1857, 557–581.

Hubert, A., 2015. Chemical and mineralogical signatures of oxygenic photosynthesis in Archean and Paleoproterozoic sediments. Thesis, Université d'Orléans.

Jeandel, C., Oelkers, E.H., 2015. The influence of terrigenous particulate material dissolution on ocean chemistry and global element cycles. Chemical Geology 395, 50–66.

Juniper, S.K., Martineu, P., Sarrazin, J., Gélinas, Y., 1995. Microbial-mineral floc associated with nascent hydrothermal activity on CoAxial Segment, Juan de Fuca Ridge. Geophysical Research Letters 22, 179-182.

Kamber, B.S., Greig, A., Collerson, K.D., 2005. A new estimate for the composition of weathered young upper continental crust from alluvial sediments, Quennsland, Australia. Geochimica et Cosmochimica Acta 69, 1041–1058.

Kamber B.S, 2015. The evolving nature of terrestrial crust from the Hadean, through the Archaean, into the Proterozoic. Precambrian Research 258, 48–82.

Kerrich, R., Said, N., Manikyamba, C., Wyman, D., 2013. Sampling oxygenated Archean hydrosphere: Implication from fractionations of Th/U and Ce/Ce* in hydrothermally altered volcanic sequences. Gondwana Research 23, 506–525.

Koeberl, C., 2006. The record of impact processes on the early Earth: A review of the first 2.5 billion years. in Reimold, W.U., and Gibson, R.L. (Eds.) Processes on the Early Earth: Geological Society of America Special Paper 405, 1–22.

Lindsay, J.F., Brasier, M.D., McLoughlin, N., Green, O., Fogel, M., Steele, A., Mertzman, S., 2005. The problem of deep carbon – an Archean paradox, Precambrian Research 143, 1–22.

Loaëc, M., Olier, R., Guezennec, J., 1998. Chelating properties of bacterial exopolysaccharides from deep-sea hydrothermal vents. Carbohydrate Polymers 35, 65–70.

Lowe, D.R., Byerly, G.R., 2007. An overview of the geology of the Barberton Greenstone Belt and vicinity: implications for early crustal development. In Van Kranendonk, M.J., Smithies, R.H., Bennett, V.H. (Eds.) Earth's Oldest Rocks, Elsevier (Developments in Precambrian Geology), Amsterdam, pp. 481-526.

Lowe, D.R., Byerly, G.R., Kyte, F.T., Shukolyukov, A., Asaro, F., Krull, A., 2003. Spherule beds 3.47–3.34 Ga-old in the Barberton greenstone belt, South Africa: A record of large meteorite impacts and their influence on early crustal and biological evolution: Astrobiology 3, 7–48.

Maurette, M., Brack, A., 2006. Cometary petroleum in Hadean time. Meteoritics and Planetary Science 41, 5247.

Maxwell, J.A., Teesdale, W.J., Campbell, J.L., 1995. The Gupix PIXE software package II. Nuclear Instruments and Method in Physics Research B 95, 407–421

McCollum TM, Ritter G, Simoneit BR. 1999. Lipid synthesis under hydrothermal conditions by Fischer-Tropsch-type reactions. Orig Life Evol Biosph 29, 153–166.

McCollum, T.M., Seewald, J.S., 2006. Carbon isotopic composition of organic compounds produced by abiotic synthesis under hydrothermal conditions. Earth and Planetary Science Letters 243, 74–84.

Moore, E.K., Jelen, B.I., Giovannelli, D., Raanan, H., Falkowski, P.G., 2017. Metal availability and the expanding network of microbial metabolisms in the Archaean eon. Nature Geoscience 10, 629–636.

Morag, N., Williford, K.H., Kitajima, K., Philippot, P., Van Kranendonk, M.J., Lepot, K., Thomazo, C., Valley, J.W., 2016. Microstructure-specific carbon isotopic signatures of organic matter from ~3.5 Ga cherts of the Pilbara Craton support a biologic origin. Precambrian Research 275, 429–449.

Nisbet, E.G., 2000. The realms of Archaean life. Nature 405, 625–626.

Nisbet, E.G., Sleep, N.H., 2001.The habitat and nature of early life. Nature 409, 1083–1091.

Orange F, Westall F, Disnar JR, Prieur D, Bienvenu N, Le Romancer M, Défarge C (2009) Experimental silicification of the extremophilic Archaea Pyrococcus abyssi and Methanocaldococcus jannaschii. Applications in the search for evidence of life in early Earth and extraterrestrial rocks, Geobiology 7, 403-418.

Orange, F., Chabin, A., Gorlas, A., Lucas-Staat, S., Geslin, C., Le Romancer, M., Prangishvili, D., Forterre, P., Westall, F., 2011. Experimental fossilisation of viruses from extremophilic Archaea. Biogeosciences 8, 1465–1475.

Schidlowski, M., 1984. Biological modulation of the terrestrial carbon cycle: Isotope clues to early organic evolution. Advances in Space Research 4, 183–193.

Schidlowski, M., 1988. A 3,800-million-year isotopic record of life from carbon in sedimentary rocks. Nature 333, 313–318.

Schultz-Lam, S., Thompson, J.B., Beveridge, T.J., 1993. Metal ion immobilization by bacterial surfaces in freshwater environments. Water Quality Research Journal 28, 51–82.

Schultz-Lam, S., Fortin, D., Davis, B.S., Beveridge, T.J., 1996. Mineralization of bacterial surfaces. Chemical Geology 132, 171–181.

Sherwood Lollar, B., Westgate, T.D., Ward, J.A., Slater, G.F., Lacrampe-Couloume, G., 2002. Abiogenic formation of alkanes in the Earth's crust as a minor source for global hydrocarbon reservoirs. Nature 416, 522–524.

Taran, Y.A., Kliger, G.A., Sevastianov, V.S., 2007. Carbon isotope effects in the open-system Fischer–Tropsch synthesis. Geochimica et Cosmochimica Acta 71, 4474–4487.

van Zuilen, M.A. Fliegel, D., Wirth, R., Lepland, A., Qu, Y., Schreiber, A., Romashkin, A.E., Philippot, P., 2012. Mineral-templated growth of natural graphite films. *Geochimica et Cosmochica Acta* **83**, 252–262.

Wacey, D., Kilburn, M.R., Saunders, M., Cliff, J., Brasier, M.D., 2011. Microfossils of sulphur-metabolizing cells in 3.4-billion-year-old rocks of Western Australia. Nature Geoscience 4, 698–702.

Wacey, D., Saunders, M., Kong, C., Brasier, A.T., Brasier, M.D., 2016. 3.46 Ga Apex chert 'microfossils' reinterpreted as mineral artefacts produced during phyllosilicate exfoliation. Gondwana Research 36, 296–313.

Wacey, D., Noffke, N., Saunders, M., Guagliardo, P., Pyle, D.M., 2018. Volcanogenic pseudo-fossils from the ~3.48 Ga Dresser Formation, Pilbara, Western Australia. Astrobiology 18, 539–555.

Westall, F., de Wit, M.J., Dann, J., van der Gaast, S., de Ronde, C.E.J., Gerneke, D., 2001. Early Archaean fossil bacteria and biofilms in hydrothermally–influenced sediments from the Barberton greenstone belt, South Africa. Precambrian Research 106, 93–116.

Westall, F., de Vries, S.T., Nijman, W., Rouchon, V., Orberger, B., Pearson, V., Watson, J., Verchovsky, A., Wright, I., Rouzaud, J.-N., Marchesini, D., and Severine, A., 2006, The 3.466 Ga "Kitty's Gap Chert," an early Archean microbial ecosystem, in Reimold, W.U., and Gibson, R.L. (Eds.) Processes on the Early Earth. Geological Society of America Special Paper 405, 105–131.

Westall, F., Foucher, F., Cavalazzi, B., de Vries, S.T., Nijman, W., Pearson, V., Watson, J., Verchovsky, A., Wright, I., Rouzaud, J.-N., Marchesini, D., Anne, S., 2011. Volcaniclastic habitats for early life on Earth and Mars: a case study from ~3.5 Ga-old rocks from the Pilbara, Australia. Planetary and Space Science 59, 1093–1106.

Westall, F., Campbell, K.A., Bréhéret, J.G., Foucher, F., Gautret, P., Hubert, A., Sorieul, S., Grassineau, N., Guido, D.M., 2015. Archean (3.33 Ga) microbe-sediment systems were diverse and flourished in a hydrothermal context. Geology.

Williams, R.J.P., 1981; The Bakerian Lecture, 1981: natural selection of the chemical elements. Proc. R. Soc. Lond. B Biol. Sci. 213, 361–397.

Williams, R.J.P., 2001. Chemical selection of elements by cells. Coordination Chemistry Reviews 216–217, 583–595.

Williams, R.J.P., Frausto da Silva, J.J.R., 2003. Evolution was chemically constrained. Journal of Theoretical Biology 220, 323–343.
